# Supplementary material for: Extracorporeal Shock Wave Therapy for Wound Management: Clinical Evidence, Energy Delivery Parameters and Mechanistic Insights—A Systematic Review
Source: J Clin Med. 2026 Nov 3;15(21):8550. doi: 10.3390/jcm15218550 (PMC13763619; doi:10.3390/jcm15218550)
Supplement: Supplementary file 1 [file jcm-15-08550-s001.zip › jcm-4623551_R2 Supplementary File S7.pdf]

Extracorporeal Shock Wave Therapy for Wound Management: Clinical Evidence, Energy Delivery Parameters and Mechanistic Insights — A Systematic Review

by Brent Musolf, Carmen Nussbaum-Krammer, Michael O'Neal, Tobias Wuerfel, Nicola Maffulli and Christoph Schmitz

Supplementary File S7

Domain-level risk-of-bias assessment (RoB 2 and ROBINS-I) of all clinical studies with a control group
(numbers in brackets refer to the reference numbers in the main text)

Note on the risk-of-bias assessment: All 26 records reporting a concurrent control group were assessed; the two reports of the same two dermaPACE trials [28,90] were assessed together, giving 25 assessments. Studies with random allocation were assessed with RoB 2 (with the additional domain for crossover trials where applicable); studies with non-random allocation (quasi-random allocation by date, odd/even number, recruitment phase or patient identification number; allocation by center or by patient choice; untreated contralateral limb as control) were assessed with ROBINS-I in its version 2 (riskofbias.info; November 2025 revision). One outcome was assessed per study: complete wound healing where reported as events and denominators in both groups, otherwise the primary wound outcome; the effect of interest was the effect of assignment to the intervention. RoB 2 overall judgments followed the standard algorithm (low risk only if all domains were at low risk; high risk if at least one domain was at high risk; otherwise some concerns). ROBINS-I overall judgments corresponded to the most severe domain judgment. Assessments were based on the published full texts and on the trial registrations cited in them. Table S7.3 gives the rationale for every domain judgment together with supporting quotations, which are reproduced verbatim from the original publications; … marks text omitted at the beginning or end of a quotation.

Summary: Of the 17 randomized trials, none was at low risk of bias overall, 13 raised some concerns and 4 were at high risk of bias. Of the 8 non-randomized controlled studies, 7 were at serious and 1 at critical risk of bias. Of the seven trials contributing to the exploratory pooled analysis (Table 11 in the main text), the five randomized trials raised some concerns and the two quasi-randomized trials [23,29] were at serious risk of bias.

Abbreviations (in alphabetical order): ABI, ankle–brachial index; ATSI, Aboriginal and Torres Strait Islander; BMI, body mass index; BWAT, Bates-Jensen Wound Assessment Tool; CCVUQ, Charing Cross Venous Ulcer Questionnaire; CI(s), confidence interval(s); CONSORT, Consolidated Standards of Reporting Trials; CRP, C-reactive protein; DFU(s), diabetic foot ulcer(s); DM, diabetes mellitus; EFD, energy flux density; EQ-5D, EuroQol five-dimension questionnaire; ESWT, extracorporeal shock wave therapy; FDA, U.S. Food and Drug Administration; fESWT, focused ESWT; GRADE, Grading of Recommendations Assessment, Development and Evaluation; HAQ, Health Assessment Questionnaire; HBO(T), hyperbaric oxygen (therapy); HR, hazard ratio; IHC, immunohistochemistry; IL-1β, interleukin-1 beta; IPC, intermittent pneumatic compression; IQR, interquartile range; ITT, intention to treat; mITT, modified intention to treat; mRSS, modified Rodnan skin score; OR(s), odds ratio(s); PAD, peripheral arterial disease; PI, principal investigator; RCA, relative change in ulcer area; RCT, randomized controlled trial; rESWT, radial ESWT; RoB 2, Cochrane risk-of-bias tool for randomized trials, version 2; ROBINS-I, Risk Of Bias In Non-randomized Studies of Interventions; SC, standard care; SD, standard deviation; SEM, standard error of the mean; SOC, standard of care; SWC, standard wound care; SWHSI, surgical wounds healing by secondary intention; SWT, shock wave

therapy; $TcPO_2$, transcutaneous partial oxygen pressure; uESWT, unfocused ESWT; VAS, visual analogue scale; WBS, Wound Bed Score.

Table S7.1. Risk of bias of the randomized controlled trials (RoB 2). D1, randomization process; D2, deviations from intended interventions; D3, missing outcome data; D4, measurement of the outcome; D5, selection of the reported result. For the crossover trial [81], the additional domain on period and carryover effects was judged high risk (see Table S7.3).

Study	Comparison	Outcome assessed	D1	D2	D3	D4	D5	Overall
**Dumfarth et al., 2008 [78]**	Single intraoperative uESWT + standard wound closure/care vs standard wound closure/care alone (no sham)	Wound healing on the ASEPSIS score, postoperative days 3-7 (primary endpoint; complete healing not reported as events)	Some concerns	Some concerns	Low	Some concerns	Some concerns	**Some concerns**
**Moretti et al., 2009 [95]**	focused electromagnetic ESWT (Minilith SL1, 3 sessions) + standard care vs standard care alone	complete healing at 20 weeks (patients): 8/15 vs 5/15 (53.3% vs 33.3%)	Some concerns	Some concerns	Low	Some concerns	Some concerns	**Some concerns**
**Larking et al., 2010 [81] (crossover)**	uESWT (Orthowave 180c, 4 weekly sessions) vs placebo head (no shock waves), sequence ESWT-first vs placebo-first, 2-week washout	Ulcer area (Visitrak tracing), change from baseline at end of each 4-week period and up to 8 weeks after end of ESWT (complete healing not reported as events per group)	Low	Low	Low	Low	Some concerns	**High**
**Ottomann et al., 2010 [82]**	Single intraoperative uESWT + standard topical therapy (Mepitel + antiseptic gel) vs standard topical therapy alone (no sham)	Time to complete (>=95%) donor-site re-epithelialization (primary endpoint; all donor sites healed within 11-20 days, so healed/not healed events are uninformative)	Low	Some concerns	Low	Low	Some concerns	**Some concerns**
**Ottomann et al., 2012 [85]**	Single uESWT after debridement + standard care (Mepitel + antiseptic gel) vs standard care alone (no sham)	Time to complete (>=95%) burn-wound epithelialization (primary endpoint; all wounds healed, so events are uninformative)	Low	Low	Low	Low	Some concerns	**Some concerns**
**Nossair et al., 2013 [110]**	Unfocused (radial) ESWT, 3 weekly sessions + medication + traditional wound care vs medication + traditional wound care alone	Wound surface area (planimetry, cm²) and epithelialization rate at 12 weeks (primary wound outcome; complete healing not reported as events)	Some concerns	Some concerns	Low	Some concerns	Some concerns	**Some concerns**
**Saggini et al., 2013 [86]**	uESWT DermaGold 0.10 mJ/mm2 (group A) vs uESWT Evotron 0.04 mJ/mm2 (group B), weekly x 7, plus same dressings	Complete wound healing at end of treatment (7 weeks), patients: group A 44/62 (text; 60 completers), group B 16/40 completers (62 randomized)	Some concerns	High	High	Some concerns	Some concerns	**High**
**Omar et al., 2014 [75]**	ESWT (8 sessions, twice weekly) + standardized wound care vs standardized wound care alone	Complete healing at 20 weeks (W20), per ulcer: 13/24 ESWT vs 6/21 control (as used in the review)	Some concerns	Some concerns	Some concerns	Low	Some concerns	**Some concerns**

Study	Comparison	Outcome assessed	D1	D2	D3	D4	D5	Overall
**Jeppesen et al., 2016 [99]**	focused electromagnetic ESWT (Duolith SD1, 6 sessions over 3 weeks) + standard care vs standard care alone	percentage ulcer area reduction at 7 weeks (primary wound outcome; complete healing not reported): 34.5% vs 5.6%, p=0.387	Low	Some concerns	Some concerns	Some concerns	Low	**Some concerns**
**Dolibog et al., 2018a [106]**	rESWT + SWC (group A, n=17) and fESWT + SWC (group B, n=15) vs SWC alone (group C, n=18)	relative percentage change in ulcer surface area at 8 weeks (4 weeks after the 6th session; primary area outcome). Complete healing was not used because SWC events are reported only graphically (Figure 3), not as numbers.	Some concerns	Some concerns	High	Some concerns	Some concerns	**High**
**Dolibog et al., 2018b [112]**	rESWT + SWC (group B, n=17) vs SWC alone (group C, n=17); ultrasound arm (group A) not considered	mean percentage reduction in ulcer area at 4 weeks (primary; no complete-healing data reported)	Some concerns	Some concerns	Low	Some concerns	Some concerns	**Some concerns**
**Snyder et al., 2018 [28]***	focused electrohydraulic ESWT (dermaPACE) + standard care vs sham ESWT + standard care	complete wound closure at 24 weeks (patients; pooled Study 1 + Study 2: 65/172 vs 43/164, all randomized in denominator)	Low	Low	Some concerns	Low	Some concerns	**Some concerns**
**Taheri et al., 2021 [102]**	focused electromagnetic ESWT (4 weekly sessions) + compression bandaging vs sham ESWT (device switched off) + compression bandaging	wound size at week 8 (primary wound outcome; complete healing not reported): 94.8 +/- 176.2 vs 307.6 +/- 433.6 (units stated cm2), time x group p=0.281	Some concerns	Some concerns	Some concerns	Some concerns	Some concerns	**Some concerns**
**Dymarek et al., 2023 [115]**	single rESWT session + SWC vs sham rESWT + SWC	change in planimetric wound area from baseline (M0) to 1 week after a single session (M1); no complete-healing data. WBS and BWAT are additional clinical scores.	High	Low	Low	Low	Some concerns	**High**
**Vangaveti et al., 2023 [119]**	ESWT every 2 weeks + standard of care vs standard of care alone	Complete healing of the index ulcer at 6 weeks (patients): 5/25 ESWT + SOC vs 6/23 SOC	Low	Some concerns	Some concerns	Some concerns	Some concerns	**Some concerns**
**Hitchman et al., 2025 [27]**	fESWT + SWC (high-dose arm 500 shocks/cm2 and low-dose arm 100 shocks/cm2, pooled) vs sham ESWT + SWC	complete healing at 24 weeks (patients); 16/25 high dose, 13/23 low dose, 11/26 sham (pooled active arms 29/48 vs 11/26)	Low	Low	Some concerns	Low	Some concerns	**Some concerns**
**Yang et al., 2025 [118]**	Radial ESWT (3 sessions, days 3, 5, 7) + routine dressing treatment vs sham ESWT (device switched off) + routine dressing treatment	Percentage wound area reduction ('percentage of wound healing') from day 1 to day 14 (primary	Some concerns	Some concerns	Low	Low	Some concerns	**Some concerns**

Study	Comparison	Outcome assessed	D1	D2	D3	D4	D5	Overall
		outcome; complete closure not reported)						

Table S7.2. Risk of bias of the non-randomized controlled studies (ROBINS-I V2). D1, confounding; D2, classification of interventions; D3, selection of participants; D4, deviations from intended interventions; D5, missing data; D6, measurement of the outcome; D7, selection of the reported result.

Study	Comparison	Outcome assessed	D1	D2	D3	D4	D5	D6	D7	Overall
**Saggini et al., 2008 [79]**	fESWT (Evotron, every 2 wk, 4-10 sessions) + continued conservative treatment vs regular conservative dressings	complete healing (ulcers) at end of treatment: ESWT 16/32 ulcers (30 patients) after 4-10 sessions (8-20 wk) vs control 1/10 ulcers (10 patients) at 8 weeks	Serious	Low	Moderate	Moderate	Low	Serious	Moderate	**Serious**
**Wang et al., 2009 [23]**	fESWT (Orthowave 180, 3 sessions over 6 wk) + wound care vs HBOT (20 daily sessions) + same wound care	complete healing (ulcers) at end of follow-up (time point not explicitly stated; mean follow-up 11.6 vs 12.1 months, range 6-14 months): 11/36 vs 8/36	Serious	Low	Low	Moderate	Low	Moderate	Moderate	**Serious**
**Wang et al., 2011 [29]**	Focused ESWT (dermaPACE, 6 sessions over 3 weeks) + standard wound care vs HBOT (20 daily sessions) + standard wound care	Complete ulcer healing after one course of treatment, per foot: ESWT 24/44 vs HBOT 10/40 (time point not precisely defined; follow-ups at 3 and 6 weeks, then 3-monthly)	Serious	Low	Low	Moderate	Moderate	Moderate	Moderate	**Serious**
**Fan et al., 2022 [25]**	Radial ESWT (Swiss DolorClast Smart, weekly for 4–6 weeks) + routine treatment and nursing intervention vs routine treatment and nursing intervention	Wound reduction rate (%) after the treatment period (4–6 weeks), the study's primary wound outcome (healing time is also reported). Complete healing is not reported as events; results appear only in Figure 1 with P<0.05, without numerical values.	Serious	Moderate	Moderate	Moderate	Moderate	Moderate	Moderate	**Serious**
**Jeong et al., 2023 [92]**	fESWT (orthoPACE, 3x/week for 3 weeks) + standard dressing/debridement on ulcerated feet vs no treatment on non-ulcerated contralateral feet	No wound healing/closure or wound area outcome reported. Primary outcome: $TcPO_2$ (mm Hg, dorsal foot) at week 3 (also weeks 1 and 2), between groups and vs baseline	Critical	Low	Low	Moderate	Low	Low	Moderate	**Critical**
**Dolibog et al., 2024 [24]**	rESWT + SC (n=16) and fESWT + SC (n=15) vs SC alone (n=18); IPC arm (n=20) not considered	relative percentage change in ulcer surface area (RCA) at 4 weeks (primary). Complete healing occurred only in the IPC arm (2 patients), i.e. no events in the ESWT and SC arms, so the primary area outcome was assessed.	Serious	Low	Moderate	Moderate	No information	Moderate	Moderate	**Serious**

Study	Comparison	Outcome assessed	D1	D2	D3	D4	D5	D6	D7	Overall
**Ishii et al., 2025 [104]**	fESWT (Duolith SD1, weekly x 8) + continued prior treatment vs conventional treatment (all available therapies)	Complete healing not reported as events per group; primary outcome assessed: mean reduction in number of digital ulcers at 8 weeks (ESWT 4.47 ± 2.65 vs conventional 0.83 ± 2.79; n = 30 vs 30)	Serious	Low	Low	Moderate	Low	Serious	Low	**Serious**
**Landscheidt et al., 2025 [105]**	focused electromagnetic ESWT (Duolith SD1, Days 5/7/9) on one half vs placebo (device off) on the other half of the same wound, both with standardized wound care	re-epithelialization (proportion of wound area epithelialized, planimetry) at Day 12, within-person comparison ESWT half (1A) vs placebo half (1B) at skin-graft recipient (chronic wound) and donor sites; donor 0.92 vs 0.82 ($p<0.05$), recipient residual area smaller ($p=0.008$)	Moderate	Low	Low	Moderate	Moderate	Moderate	Serious	**Serious**

Table S7.3. Rationale and supporting quotations for each domain judgment.

Dumfarth et al., 2008 [78] — RoB 2

Design as reported: Prospective randomized trial (single center), prophylactic SWT after vein harvesting for CABG. Design as assessed: RCT (parallel). Comparison: Single intraoperative uESWT + standard wound closure/care vs standard wound closure/care alone (no sham). Outcome: Wound healing on the ASEPSIS score, postoperative days 3-7 (primary endpoint; complete healing not reported as events). Participants: 100 randomized (50 SWT / 50 control); tables report 50/50.

Domain	Judgment	Rationale	Supporting quotations
D1 Randomization process	**Some concerns**	Random allocation (1:1, stratified by age, sex, BMI) is stated but the sequence generation method and allocation concealment are not described. Baseline characteristics and operative data are comparable.	*"Patients were randomly allocated to the…"*<br>*"SWT group and control group in a 1:1 ratio stratified accord-…"*<br>*"…trol group were comparable according to age, sex, body…"*
D2 Deviations from intended interventions	**Some concerns**	Patients were anesthetized during the single intraoperative application, but surgical/ward personnel were not blinded and there was no sham; no information on deviations or co-interventions arising from trial context. Analysis appears to include all 100 randomized patients.	*"…treatment group, low-energy SWT was applied after wound…"*<br>*"…the control group underwent routine vein graft harvesting…"*
D3 Missing outcome data	**Low**	Results tables report n = 50 per group, i.e. apparently all randomized patients. Three in-hospital deaths (1 SWT, 2 control) occurred, timing relative to days 3-7 not given, but numbers are too small to bias the result materially.	*"…in-hospital deaths (3% of the study population), 2 in the…"*
D4 Measurement of the outcome	**Some concerns**	The ASEPSIS score was quantified by a blinded investigator, but it includes points for additional treatment (antibiotics, drainage, debridement) and prolonged stay, which were decided by treating clinicians whose blinding is not reported; the antibiotic component (4% vs 22%) contributes heavily to the score difference.	*"…was quantified by a blinded investigator from postoper-…"*<br>*"Additional treatment:"*
D5 Selection of the reported result	**Some concerns**	No protocol or trial registration is mentioned. The ASEPSIS score was analyzed in two ways (daily scores and categories); both are reported and point in the same direction, so nothing clearly suspicious, but pre-specification cannot be verified.	*"The ASEPSIS score was analyzed by two methods. First,…"*<br>*"A Bonferoni-Holm correction for multiple…"*
**Overall**	**Some concerns**	Some concerns in D1, D2, D4, D5 and no domain at high risk, so the overall judgment is Some concerns (standard RoB 2 algorithm; no escalation for multiple Some concerns).	

Saggini et al., 2008 [79] — ROBINS-I V2

Design as reported: Preliminary prospective study of consecutive patients treated with ESWT, compared with a control group of 10 consecutive patients treated with regular dressings. Design as assessed: cohort with concurrent (non-randomized) controls. Comparison: fESWT (Evotron, every 2 wk, 4-10 sessions) + continued conservative treatment vs regular conservative dressings. Outcome: complete healing (ulcers) at end of treatment: ESWT 16/32 ulcers (30 patients) after 4-10 sessions (8-20 wk) vs control 1/10 ulcers (10 patients) at 8 weeks. Participants: 40 patients (30 ESWT with 32 ulcers; 10 controls with 10 ulcers); all analyzed.

Domain	Judgment	Rationale	Supporting quotations
D1 Bias due to confounding	**Serious**	Patients offered and accepting ESWT formed the treatment group. How the 10 controls were chosen ('randomly recruited') is not explained. The case mix differed on a strong prognostic factor: 16/32 ESWT ulcers were posttraumatic, the type the authors say responded best, vs 2/10 controls, and controls were older (mean 66.6 vs 58.5 y). There was no adjustment for any confounder.	*"…were counseled about the use of ESWT as alternative…"*<br>*"A group of 10 consecutive patients, randomly re-…"*<br>*"This was more noticeable in the treatment of post-…"*<br>*"…traumatic chronic ulcers than in the venous or diabetic…"*
D2 Bias in classification of interventions	**Low**	Intervention status (ESWT vs dressings only) was clear and set at the start of treatment.	*"…were treated with conservative dressings representing the…"*
D3 Bias in selection of participants into the study (or into the analysis)	**Moderate**	ESWT patients joined 'after a strict selection', but the selection criteria beyond eligibility are not reported. Recruitment of controls (setting, timing, start of follow-up) is not described. There is no clear evidence of selection based on post-intervention characteristics.	*"…joined the study after a strict selection and consent/…"*<br>*"During the study period, 40 consecutive patients…"*
D4 Bias due to deviations from intended interventions	**Moderate**	The study was open-label. The number of ESWT sessions (4-10) depended on response, and both groups continued conservative care, which is not described in detail. One ESWT patient later needed surgery for osteomyelitis. Deviations and co-interventions are not otherwise reported.	*"…weeks, with a minimum of four and a maximum of 10…"*<br>*"ESW treatment sessions, the patients continued the pre-…"*
D5 Bias due to missing data	**Low**	Individual outcome data are tabulated for all 32 ESWT ulcers and all 10 control ulcers. No losses are reported.	*"At the end of the study period, 16 ulcers healed…"*<br>*"In the control group, only one wound completely…"*
D6 Bias in measurement of the outcome	**Serious**	Assessors were not blinded (photographic and clinical assessment by the treating team). Outcome timing also differed by group: controls were assessed at 8 weeks, while ESWT ulcers were followed to the end of treatment (up to 10 sessions, about 20 weeks). The longer observation window favors ESWT for complete healing.	*"…healed after eight weeks (Table 6)"*<br>*"…within the first fourth to sixth sessions."*<br>*"Final clinical result after 10 sessions (20 wk) of ESWT…"*
D7 Bias in selection of the reported result	**Moderate**	No protocol, registration or pre-specified primary outcome is reported. Complete healing was never formally compared between groups. The only between-group test (Wilcoxon matched-pairs on differences) covers area, granulation tissue and pain, and the ESWT area results are reported for non-healed wounds only. Several outcomes and subgroups are reported selectively by ulcer type.	*"Wilcoxon matched pairs rank sum test applied on…"*<br>*"…differences between the two groups showed a statistical…"*
**Overall**	**Serious**	D1 (non-comparable self-selected groups with no adjustment) and D6 (unblinded assessment over different observation windows: 8 wk vs up to 20 wk) are Serious, so the overall judgment is Serious; D1 is borderline Critical given the imbalance in posttraumatic ulcers.	

Moretti et al., 2009 [95] — RoB 2

Design as reported: Randomized, prospective, controlled clinical trial (single center, Bari, Italy); registered ISRCTN21800909. Design as assessed: RCT (parallel). Comparison: focused electromagnetic ESWT (Minilith SL1, 3 sessions) + standard care vs standard care alone. Outcome: complete healing at 20 weeks (patients): 8/15 vs 5/15 (53.3% vs 33.3%). Participants: 30 randomized (15 vs 15); 30 analyzed.

Domain	Judgment	Rationale	Supporting quotations
D1 Randomization process	**Some concerns**	The paper only states that patients were randomized; neither the sequence-generation method nor allocation concealment is described. Baseline age and ulcer area are reported as not significantly different (ESWT ulcers somewhat larger, 297.8 vs 245 mm2; sex 9/6 vs 7/8), with no sign of a problem with randomization.	*"The population was randomized i nto two groups that…"*<br>*"Th is study is a ra ndomized, prospected, control led…"*<br>*"There were no sign ificant differences between the two…"*
D2 Deviations from intended interventions	**Some concerns**	No sham; patients and treating clinicians were aware of allocation (ESWT applied with audible device versus standard care alone). No information on deviations from intended interventions or co-interventions arising from the trial context; all randomized patients were analyzed in their allocated group (no exclusions), so the analysis was appropriate.	*"Patients in the control group were treated with the essen…"*<br>*"A.I I patients of both groups com pleted the study and…"*
D3 Missing outcome data	**Low**	All 30 randomized patients completed the study and attended all visits; individual outcome data are tabulated for every patient.	*"A.I I patients of both groups com pleted the study and…"*<br>*"…attended aJI con trol visits."*
D4 Measurement of the outcome	**Some concerns**	Healing was documented with digital photographs and computer planimetry (Rhinoceros), but who judged complete healing and whether the assessor was blinded is not reported; in an open trial the assessor was probably aware of allocation. Complete closure is a fairly objective endpoint, so influence is possible but not likely to be large.	*"The u lcers were photographed by digital camera usi ng the…"*
D5 Selection of the reported result	**Some concerns**	An ISRCTN number is cited, but the ISRCTN record (checked online in the second review) shows the trial was submitted on 02/07/2008 and registered on 29/09/2008, i.e. after recruitment and completion (October 2006 - March 2007): retrospective registration. The registered primary outcomes are wound-area reduction (Rhinoceros planimetry), time to complete healing and a re-epithelialization index, not the proportion healed at 20 weeks. Nothing clearly suggests selective reporting, but pre-specification cannot be confirmed, so Some concerns, consistent with the other retrospectively registered trials (Ottomann et al., 2012 [85], Taheri et al., 2021 [102], Omar et al., 2014 [75], Yang et al., 2025 [118]).	*"Trial registration: Current Controlled Trials ISRCTN2 I 800909…"*<br>*"…between October J , 2006 and March 3 1 ,…"*<br>*"…and B groups were 5 3 .33% and 3 3 . 3 3%, respectively."*
**Overall**	**Some concerns**	Some concerns in D1, D2, D4, D5 and no domain at high risk, so the overall judgment is Some concerns (standard RoB 2 algorithm; no escalation for multiple Some concerns).	

Wang et al., 2009 [23] — ROBINS-I V2

Design as reported: Prospective study, patients 'randomly divided' into two groups (presented as randomized). Design as assessed: quasi-randomized (allocation by odd/even day of referral/treatment). Comparison: fESWT (Orthowave 180, 3 sessions over 6 wk) + wound care vs HBOT (20 daily sessions) + same wound care. Outcome: complete healing (ulcers) at end of follow-up (time point not explicitly stated; mean follow-up 11.6 vs 12.1 months, range 6-14 months): 11/36 vs 8/36. Participants: 74 patients / 76 ulcers allocated (36/38 ESWT, 38/38 HBO); 70 patients / 72 ulcers analyzed (34/36 ESWT, 36/36 HBO).

Domain	Judgment	Rationale	Supporting quotations
D1 Bias due to confounding	**Serious**	Allocation followed the day of referral (odd vs even days), not a random sequence; the rule was predictable and there was no allocation concealment, so referring clinicians could influence who got which treatment. There was no adjustment for any confounder. Measured baseline variables are broadly similar, but ESWT patients were younger (58.6 vs 63.4 y, P = 0.072) and ulcer location differed (dorsal 23 vs 17). Judged Serious for consistency with Wang et al., 2011 [29] (same group, same odd/even rule, same HBOT comparator, no adjustment): under ROBINS-I V2 an unadjusted comparison with predictable allocation does not control confounding.	*"…were randomly divided into two groups according to the dates of…"*<br>*"Thirty-six patients with 38 ulcers were referred on odd…"*<br>*"…patients with 38 ulcers were referred on even days of the week and…"*<br>*"Average age (y) 58.6…"*
D2 Bias in classification of interventions	**Low**	The two interventions are distinct and were set at allocation by the date rule, so they could not be misclassified.	—
D3 Bias in selection of participants into the study (or into the analysis)	**Low**	Consecutive eligible patients were enrolled over a defined period and allocated at the start of treatment, so start of follow-up and start of intervention coincide. Post-allocation exclusions are covered under D5.	*"August 2006 and January 2007, 74 patients with 76 chronic…"*
D4 Bias due to deviations from intended interventions	**Moderate**	Neither patients nor personnel were blinded. Repeat ESWT courses were allowed for incompletely healed ulcers, with no equivalent in the HBO arm. Patients with poor compliance were dropped, so the analysis was per protocol rather than ITT. Background wound care was stated to be the same in both arms, and there is no other information on deviations.	*"…of three treatments in 6 wk. A repeat course of treatment was…"*<br>*"…performed in cases with incomplete healing from the first course of…"*<br>*"Patients received the same wound care as the ESWT group."*
D5 Bias due to missing data	**Low**	4 of 74 patients (5%; 2 per arm) were excluded for poor compliance. That is small, balanced, and unlikely to change the healing comparison materially, although these patients are missing from an ITT analysis.	*"…received HBO therapy. During the course of treatment, four patients…"*<br>*"…with four ulcers were excluded because of poor compliance including…"*
D6 Bias in measurement of the outcome	**Moderate**	Healing was judged by visual examination and photographs. Only the pathologists (histology/IHC) are described as blinded, so clinical assessors presumably knew the allocation. Complete epithelialization is a fairly hard endpoint but still needs judgment, and the assessment time point is not defined. (Moderate is applied uniformly to unblinded visual/photographic assessment of wound closure across the ROBINS-I studies.)	*"The extent of wound healing with…"*<br>*"…epithelization at different time intervals was documented with vi-…"*<br>*"Two pathologists blinded to the treatment…"*
D7 Bias in selection of the reported result	**Moderate**	No protocol, registration or pre-specified analysis plan is mentioned. The healing outcome is reported only as a 3-category result (healed / >=50% improved / unchanged) with one overall chi-square p value, the time point is not given, and the paper reports several outcomes. Nothing clearly shows selective reporting.	*"…results between the ESWT group and the HBO group were compared…"*

Domain	Judgment	Rationale	Supporting quotations
**Overall**	**Serious**	D1 is Serious (predictable odd/even-day allocation without concealment and no adjustment for confounders), so the overall judgment is Serious; other domains are Low or Moderate.	

Larking et al., 2010 [81] — RoB 2 (crossover)

Design as reported: Double-blind randomized cross-over study (ulcers randomized). Design as assessed: RCT (crossover), ulcer-level randomization (8 patients/9 ulcers; one patient contributed two ulcers). Comparison: uESWT (Orthowave 180c, 4 weekly sessions) vs placebo head (no shock waves), sequence ESWT-first vs placebo-first, 2-week washout. Outcome: Ulcer area (Visitrak tracing), change from baseline at end of each 4-week period and up to 8 weeks after end of ESWT (complete healing not reported as events per group). Participants: 9 ulcers in 8 patients randomized (4 ESWT-first, 5 placebo-first); all analyzed.

Domain	Judgment	Rationale	Supporting quotations
D1 Randomization process	**Low**	Allocation of sequence by shuffled cards in sealed envelopes, which makes the next allocation unpredictable; not stated whether envelopes were opaque/numbered. In a crossover, baseline imbalance between sequences is of limited concern (baseline areas 1.79 vs 1.23 cm2).	*"Randomization was by shuffled cards in sealed…"*<br>*"All ulcers had equal opportunity of…"*
DS Bias arising from period and carryover effects	**High**	Only a 2-week washout although the authors themselves describe a delayed, sustained ESWT effect starting 6-8 weeks after the first session; the placebo period of the ESWT-first sequence is therefore contaminated, and no test or adjustment for carryover/period effects was made.	*"…followed by a two-week…"*<br>*"…ulcers showed improved healing starting 6–8 weeks after the start of extracorporeal…"*<br>*"…delayed and sustained effect of extracorporeal…"*
D2 Deviations from intended interventions	**Low**	Placebo head on the same machine producing the same noise; usual dressing regime unchanged; no withdrawals or deviations reported. (The non-standard analysis is addressed under DS and D5.)	*"…and the other which did not (placebo) though the…"*<br>*"…wound (i.e. the patient treatment was not changed)."*
D3 Missing outcome data	**Low**	All randomized ulcers completed the study; none withdrawn.	*"All the patients who met the criteria were included…"*<br>*"…and none were withdrawn (Figure 1)."*
D4 Measurement of the outcome	**Low**	Area measured by tracing and computerized grid (average of three measurements) by an assessor blinded to treatment/placebo.	*"The assessor carrying out the measurements was…"*<br>*"…blind to which form of treatment/placebo was…"*<br>*"…system (Visitrak; Smith & Nephew, London,…"*
D5 Selection of the reported result	**Some concerns**	An ISRCTN number is cited, but no pre-specified analysis is reported; results are presented as within-sequence changes from baseline at multiple time points with selective significance markers rather than a within-ulcer ESWT-vs-placebo comparison. The flowchart labels contradict the text (group A described as ESWT-first in text but placebo-first in Figure 1).	*"…(ISRCTN88965832)."*<br>*"Ulcer mean size difference (cm2) from baseline across timepoints…"*<br>*"Group A: No. ulcers placebo first=5…"*
**Overall**	**High**	High because at least one domain is High (DS); some concerns also in D5 (standard RoB 2 algorithm).	

Ottomann et al., 2010 [82] — RoB 2

Design as reported: Prospective randomized trial (CONSORT), split-thickness skin graft donor sites; stopped early at interim analysis. Design as assessed: RCT (parallel). Comparison: Single intraoperative uESWT + standard topical therapy (Mepitel + antiseptic gel) vs standard topical therapy alone (no sham). Outcome: Time to complete (>=95%) donor-site re-epithelialization (primary endpoint; all donor sites healed within 11-20 days, so healed/not healed events are uninformative). Participants: 28 randomized (13 ESWT / 15 control); 28 analyzed (planned 27 per group; stopped early).

Domain	Judgment	Rationale	Supporting quotations
D1 Randomization process	**Low**	Computer-generated sequence; sequence concealed until assignment; no baseline imbalance of concern.	*"The randomization sequence was computer…"*<br>*"…quence was concealed until the treatment group was as-…"*<br>*"Statistical tests indicated no unbalanced distribution of subject characteristics across the two…"*
D2 Deviations from intended interventions	**Some concerns**	Patients were blinded (treated under anesthesia), but the treating physician was aware of allocation and no sham was used; no information on deviations from intended co-interventions (daily dressing changes). All randomized patients were analyzed.	*"…the patients were blinded to treatment allocation…"*<br>*"The treating physician was aware of…"*<br>*"All 28 subjects were available for…"*
D3 Missing outcome data	**Low**	Outcome data available for all 28 randomized patients.	*"All 28 subjects were available for…"*
D4 Measurement of the outcome	**Low**	Completeness of epithelialization determined from serial calibrated digital images by an independent expert blinded to group.	*"…donor sites were reviewed by an expert in wound care who…"*<br>*"…was blinded to treatment group assignment, and who de-…"*
D5 Selection of the reported result	**Some concerns**	No trial registration or published protocol cited (only ethics approval). Primary outcome and an interim analysis were pre-specified in the Methods, and the primary outcome is reported as planned; however the trial was stopped early for benefit at the interim analysis (28 of 54 planned), and the planned secondary outcomes are not reported.	*"…primary outcomes analysis was planned midway through…"*<br>*"This study was terminated early,…"*
**Overall**	**Some concerns**	Some concerns in D2, D5 and no domain at high risk, so the overall judgment is Some concerns (standard RoB 2 algorithm; no escalation for multiple Some concerns).	

Wang et al., 2011 [29] — ROBINS-I V2

Design as reported: Prospective open-label, randomized, not blinded study (ESWT vs HBOT). Design as assessed: quasi-randomized (odd/even number allocation, described by the authors as predictable). Comparison: Focused ESWT (dermaPACE, 6 sessions over 3 weeks) + standard wound care vs HBOT (20 daily sessions) + standard wound care. Outcome: Complete ulcer healing after one course of treatment, per foot: ESWT 24/44 vs HBOT 10/40 (time point not precisely defined; follow-ups at 3 and 6 weeks, then 3-monthly). Participants: 86 patients/93 feet allocated (41/46 ESWT, 45/47 HBOT); 77 patients/84 feet analyzed (39/44 vs 38/40).

Domain	Judgment	Rationale	Supporting quotations
D1 Bias due to confounding	**Serious**	Allocation by odd/even numbers, which the authors call predictable, so selective enrollment/allocation cannot be excluded. Groups differ in key prognostic factors favoring ESWT (median ulcer size 4 vs 7 cm2, P = 0.059; ABI 1.07 vs 0.91, P = 0.060; different duration ranges); no adjustment for confounders.	*"This is a prospective open-label, randomized, but not…"*<br>*"46 feet with odd numbers were assigned to the ESWT group."*<br>*"…patients were randomized in a predictable way, and both…"*<br>*"4 (1.5–9)"*<br>*"7 (2–12)"*
D2 Bias in classification of interventions	**Low**	Interventions were distinct (ESWT vs HBOT) and assigned at baseline; classification not influenced by outcome.	*"Forty-five patients with 47 feet with even numbers were…"*
D3 Bias in selection of participants into the study (or into the analysis)	**Low**	Consecutive enrollment with start of follow-up at start of treatment. The risk that predictable allocation influenced enrollment is assessed under D1 (as in Wang et al., 2009 [23]), and the exclusion of patients 'lacking complete follow-up data' is a post-allocation loss assessed under D5, so it is not counted twice here. Ten patients (4 vs 6) entered only after debridement had stabilized the ulcer, a pre-treatment step applied in both groups (Wang et al., 2009 [23] did the same and was rated Low).	*"…lacking complete follow-up data."*<br>*"…patients (4 in the ESWT group and 6 in the HBOT group)"*
D4 Bias due to deviations from intended interventions	**Moderate**	Open-label; additional antibiotics and a second treatment course were at the treating physician's discretion (second course in 14 vs 17 feet). The assessed outcome is after the first course, but co-intervention balance is not reported.	*"…administration of additional antibiotic was at the discretion of…"*<br>*"…weeks from the first treatment. The decision for a second…"*
D5 Bias due to missing data	**Moderate**	9/86 patients (2 ESWT, 7 HBOT; 9/93 feet) excluded for poor compliance or incomplete follow-up; differential (5% vs 16%) and no sensitivity analysis, although even if all missing HBOT feet had healed the direction of effect would persist.	*"9 patients were excluded including 2 in the ESWT group…"*<br>*"…(2 poor compliance) and seven in the HBO group (7 incomplete…"*
D6 Bias in measurement of the outcome	**Moderate**	Complete healing was judged by physical examination, visual observation and photographs by unblinded personnel (the ESWT operator also did follow-up photo-documentation), and the time point of 'after one course' is not precisely defined. Complete epithelialization is a relatively hard endpoint and the same method was used in both groups; Moderate is applied for consistency with Wang et al., 2009 [23] and the other ROBINS-I studies with unblinded wound-closure assessment. The longer mean follow-up in the ESWT group (13.5 vs 11.1 months) does not affect the 'after one course' outcome.	*"…ulcer status was performed by physical examination including…"*<br>*"…patients and healthcare providers were not blinded to the…"*<br>*"…tion on follow-up examinations, case review and proof read…"*
D7 Bias in selection of the reported result	**Moderate**	A registration number is cited, but it appears to have been obtained close to manuscript submission (retrospective; to be verified). Outcome categories (healed/50% improved/unchanged) are reported after both courses; no evidence of selective reporting among several outcome measures, but pre-specification cannot be confirmed.	*"…this study is NCT01219127. The inclusion criteria included…"*<br>*"…outcomes of 85% and 50% for ESWT and HBOT respectively in…"*

Domain	Judgment	Rationale	Supporting quotations
**Overall**	**Serious**	D1 is Serious (predictable odd/even allocation, prognostic imbalance in ulcer size and ABI favoring ESWT, no adjustment), so the overall judgment is Serious; all other domains are Low or Moderate.	

Ottomann et al., 2012 [85] — RoB 2

Design as reported: Prospective randomized phase II trial (CONSORT), superficial second-degree burns; predefined cohort of a larger 100-patient study. Design as assessed: RCT (parallel). Comparison: Single uESWT after debridement + standard care (Mepitel + antiseptic gel) vs standard care alone (no sham). Outcome: Time to complete (>=95%) burn-wound epithelialization (primary endpoint; all wounds healed, so events are uninformative). Participants: 50 randomized (group sizes at randomization not reported); 44 analyzed (22/22); 6 excluded (1 incomplete data, 5 lost to follow-up).

Domain	Judgment	Rationale	Supporting quotations
D1 Randomization process	**Low**	Computer-generated sequence (Rancode), concealed until assignment. Marked age imbalance among analyzed patients (52.5 vs 37.5 years, P = 0.002) and more cardiovascular disease in the ESWT group, but with adequate concealment this is compatible with chance, works against ESWT, and was adjusted for in a covariate analysis.	*"Patients were randomized in a 1:1 ratio using Rancode 3.6…"*<br>*"…random number generation. The randomization sequence was con-…"*<br>*"…(P > 0.05) except for older age (53 ± 17 vs. 38 ± 13 years, P = 0.002) in…"*
D2 Deviations from intended interventions	**Low**	Participants blinded; personnel blinding not reported (no sham) but the authors state all patients were treated as randomized, so deviations from intended interventions are unlikely. The 'intent-to-treat' analysis excluded 6 patients - handled under D3.	*"…cealed until study group assignment. Study participants were blinded…"*<br>*"…were treated as randomized. Randomization was achieved through a…"*
D3 Missing outcome data	**Low**	6/50 (12%) excluded (5 lost to follow-up, 1 incomplete data); distribution by arm not reported. A sensitivity analysis imputing an unfavorable scenario (13 days for missing ESWT, 8 days for missing controls) retained a significant 2.0-day difference, providing evidence the result is not materially biased by missingness.	*"…incomplete data (1 patient) or loss to follow up (5 patients), leaving…"*<br>*"…the healing time was set to 13 days for those…"*<br>*"ESWT patients with missing data and was set to 8 days for the control…"*
D4 Measurement of the outcome	**Low**	Time to >=95% epithelialization determined from serial digital images by two independent reviewers blinded to group (ICC 0.986).	*"Serial digital images of study wounds were reviewed by an…"*<br>*"…expert in wound care, blinded to treatment group assignment, who…"*<br>*"…lation was 0.986 for the time to ≥95% reepithelialization."*
D5 Selection of the reported result	**Some concerns**	A ClinicalTrials.gov number is cited, but the trial (Dec 2006-Dec 2007) appears to have been registered retrospectively (NCT0124xxxx numbers date from late 2010); sample size set without a formal power calculation; the cohort is described as a 'predefined' subset of a larger 100-patient study. The primary outcome is consistent with the stated one and the adjusted and sensitivity analyses agree, so nothing clearly selective, but pre-specification cannot be verified.	*"…icalTrials.gov identifier: NCT01242423)."*<br>*"…(SD) for the time to ≥95% reepithelialization; no formal power…"*<br>*"…follow-up) with acute second-degree burns from a larger study of 100 patients…"*
**Overall**	**Some concerns**	Some concerns in D5 and no domain at high risk, so the overall judgment is Some concerns (standard RoB 2 algorithm; no escalation for multiple Some concerns).	

Nossair et al., 2013 [110] — RoB 2

Design as reported: Randomized controlled trial ("divided randomly into two groups"), parallel, two arms. Design as assessed: RCT (parallel). Comparison: Unfocused (radial) ESWT, 3 weekly sessions + medication + traditional wound care vs medication + traditional wound care alone. Outcome: Wound surface area (planimetry, cm^2) and epithelialization rate at 12 weeks (primary wound outcome; complete healing not reported as events). Participants: 40 randomized (20 ESWT, 20 control); 40 analyzed.

Domain	Judgment	Rationale	Supporting quotations
D1 Randomization process	**Some concerns**	Random allocation is stated but the method of sequence generation and any allocation concealment are not described. Baseline age and wound surface area were similar between groups, so nothing suggests a problem with randomization.	*"…ulcers were randomly divided into two groups (shock wave group and…"*<br>*"…showed that there is no significant difference between…"*
D2 Deviations from intended interventions	**Some concerns**	No sham and no blinding of participants or personnel (ESWT added to wound care vs wound care alone), and nothing is reported on deviations from intended interventions or on co-interventions. All randomized patients completed the study and were analyzed, so the analysis was appropriate for the assignment effect.	*"…the control group received traditional wound care."*<br>*"…continued the study until the end of it. None refused…"*
D3 Missing outcome data	**Low**	All 40 randomized patients completed follow-up; no withdrawals.	*"All the patients involved in the study have been…"*<br>*"…continued the study until the end of it. None refused…"*
D4 Measurement of the outcome	**Some concerns**	Wound area was measured by manual acetate tracing and counting of graph-paper squares, an appropriate method applied identically in both groups, but nothing indicates that the assessor was blinded. Wound tracing of the perimeter involves some judgment, which knowledge of assignment could influence.	*"It was calculated according to planimeter…"*<br>*"…on the film with fine tipped transparency marker."*
D5 Selection of the reported result	**Some concerns**	No trial registration or pre-specified analysis plan is mentioned (only ethics approval of 'the protocol'). The two outcomes named in the methods are both reported at the single time point, so nothing suggests selective reporting; however, the reported statistics are internally inconsistent (see notes).	*"…of assessment were wound surface area and epithelialization rate."*<br>*"The protocol of this study approved by the…"*
**Overall**	**Some concerns**	Some concerns in D1, D2, D4, D5 and no domain at high risk, so the overall judgment is Some concerns (standard RoB 2 algorithm; no escalation for multiple Some concerns).	

Saggini et al., 2013 [86] — RoB 2

Design as reported: Randomized blind-assessment controlled trial comparing two unfocused ESWT devices/energy levels. Design as assessed: RCT (parallel), active-active comparison (no non-ESWT control). Comparison: uESWT DermaGold 0.10 mJ/mm2 (group A) vs uESWT Evotron 0.04 mJ/mm2 (group B), weekly x 7, plus same dressings. Outcome: Complete wound healing at end of treatment (7 weeks), patients: group A 44/62 (text; 60 completers), group B 16/40 completers (62 randomized). Participants: 124 randomized (62/62); 100 analyzed (60/40); 2 vs 22 withdrawals.

Domain	Judgment	Rationale	Supporting quotations
D1 Randomization process	**Some concerns**	Only 'randomly divided' is stated; no sequence generation or concealment information. Sex distribution differs (39M/23F vs 28M/34F) and ulcer etiology mix differs; baseline for group B is reported only for completers.	*"…therapy (mean 10 months). They were randomly divided…"*<br>*"Sixty-two subjects (39 men and 23 women) aged…"*<br>*"Sixty-two subjects (28 men and 34 women) aged…"*
D2 Deviations from intended interventions	**High**	Participants and therapists were not blinded (two different devices). 22/62 (35%) of group B stopped after the first session (15 because of pain) and were excluded from the final evaluation; analysis was per-protocol/completers only, with a substantial, imbalanced exclusion that could affect the result.	*"…of whom dropped out of the study after the first treatment…"*<br>*"…session and were not included in the final evaluation."*<br>*"…from 15 patients who withdrew from the study…"*
D3 Missing outcome data	**High**	Outcome missing for 2/62 (A) and 22/62 (B); no sensitivity analysis. Withdrawals were related to treatment pain and difficulty moving, so missingness plausibly depends on the true outcome; reporting also inconsistent (44 + 18 = 62 healed/partial in group A although 2 dropped out).	*"…were included in Group B, 22…"*<br>*"After 7 weeks of treatment 44 patients showed a…"*<br>*"…complete wound healing while the other 18 had a…"*
D4 Measurement of the outcome	**Some concerns**	The design is labeled 'blind assessment' and wounds were photo-documented, but who assessed, how blinding was achieved, and the definition of complete healing are not described.	*"A randomized blind assessment controlled trial was…"*<br>*"…morphological evolution was monitored, taking photos…"*
D5 Selection of the reported result	**Some concerns**	No protocol or registration. The stated primary outcome is percentage area reduction; complete healing counts are reported only in the narrative, with inconsistent denominators; no between-group test for complete healing.	*"…of the percentage of wound healing (calculated as the…"*<br>*"At the end of therapy, 1 6 patients achieved…"*
**Overall**	**High**	High because at least one domain is High (D2, D3); some concerns also in D1, D4, D5 (standard RoB 2 algorithm).	

Omar et al., 2014 [75] — RoB 2

Design as reported: Single-blinded (assessor) randomized controlled trial, parallel, two arms. Design as assessed: RCT (parallel); patients randomized, outcomes analyzed per ulcer (45 ulcers in 38 patients). Comparison: ESWT (8 sessions, twice weekly) + standardized wound care vs standardized wound care alone. Outcome: Complete healing at 20 weeks (W20), per ulcer: 13/24 ESWT vs 6/21 control (as used in the review). Participants: 44 patients / 52 ulcers randomized (text: 22 vs 22 patients; flow chart: 21 patients/27 ulcers vs 23 patients/25 ulcers); 38 patients / 45 ulcers analyzed (19/24 vs 19/21).

Domain	Judgment	Rationale	Supporting quotations
D1 Randomization process	**Some concerns**	The sequence was computer-generated with block labels, but allocation concealment is not described. Baseline characteristics of the analyzed patients are balanced. However, the text and the flow chart give different allocation numbers (22/22 vs 21/23 patients), which is unexplained.	*"Randomization was performed using the computer generator…"*<br>*"…block labels describing the treatment groups."*<br>*"…assigned into; control-group (n = 22) and ESWT-group (n = 22)."*<br>*"21 patients/27 ulcers…"*
D2 Deviations from intended interventions	**Some concerns**	Only the outcome assessor was blinded; patients and treating therapists knew the assignment, and no sham was used. Nothing is reported on deviations arising from the trial context, and the Discussion says 6–8 rather than 8 sessions were given. The analysis was per protocol: 6 of 44 randomized patients (7/52 ulcers) were excluded, including 3 excluded after they developed infection. A worst-case reallocation (13/27 vs 6/25) would not change the direction of the effect, so the impact is probably not substantial. The ulcer-level analysis ignores clustering of ulcers within patients (7 patients with >1 ulcer); this is a unit-of-analysis issue that affects precision rather than bias.	*"Blinded therapist evaluated the patients and measured wound…"*<br>*"…three patients (one in the ESWT-group and two in the control-…"*<br>*"…patients were excluded because of the development of…"*<br>*"…were included in the final analysis."*
D3 Missing outcome data	**Some concerns**	Outcome data are missing for 7 of 52 ulcers (3/27 vs 4/25; 14% of patients). No sensitivity analysis was done, and missingness may depend on the true outcome: 3 patients were excluded for infection, which predicts non-healing. Proportions and reasons are similar across arms, so a substantial bias is not likely.	*"2 patients/3 ulcers…"*<br>*"…infection (one in the ESWT-group and two in the control-…"*<br>*"…were included in the final analysis."*
D4 Measurement of the outcome	**Low**	Complete healing had a clinical definition (100% re-epithelialization, no drainage or dressing) and was assessed by a blinded therapist, using the same method in both groups.	*"Blinded therapist evaluated the patients and measured wound…"*<br>*"The wounds were labeled completely healed only if they…"*<br>*"…clinically closed (100% re-epithelization) without drainage or…"*
D5 Selection of the reported result	**Some concerns**	A registration (ACTRN12613000355774) is cited, but the 2013 number suggests it was registered close to the end of recruitment (May 2011 – April 2013), i.e. retrospectively. Complete healing is reported at both time points (W8, W20) without evidence of selection, but the registered primary outcome cannot be verified from the paper.	*"…code of this study was ACTRN12613000355774."*<br>*"…from May 2011 to April 2013."*
**Overall**	**Some concerns**	Some concerns in D1, D2, D3, D5 and no domain at high risk, so the overall judgment is Some concerns (standard RoB 2 algorithm; no escalation for multiple Some concerns).	

Jeppesen et al., 2016 [99] — RoB 2

Design as reported: Open-label randomized clinical trial (single center, Odense, Denmark; explorative); NCT02251418. Design as assessed: RCT (parallel). Comparison: focused electromagnetic ESWT (Duolith SD1, 6 sessions over 3 weeks) + standard care vs standard care alone. Outcome: percentage ulcer area reduction at 7 weeks (primary wound outcome; complete healing not reported): 34.5% vs 5.6%, p=0.387. Participants: 23 randomized (11 vs 12); 21 analyzed (10 vs 11).

Domain	Judgment	Rationale	Supporting quotations
D1 Randomization process	**Low**	Computer-generated sequence in REDCap in blocks of six, generated by a hospital employee not otherwise involved; block size concealed; allocation released by the software only after all baseline variables were recorded, so concealment was adequate. Several baseline imbalances (sex 45% vs 92% male, Charlson >=5 18% vs 58%, diabetes duration, creatinine, CRP, ulcer duration) are large but plausible by chance with 23 patients and point in different directions; they do not indicate a failure of randomization.	*"…intervention group or a control group in blocks of six."* *"REDCap27 and allocation of patients was done by the…"* *"…software after all baseline variables had been collected."* *"…involved in the project generated the allocation…"* *"As a consequence of imbalanced baseline variables,…"*
D2 Deviations from intended interventions	**Some concerns**	Open-label without sham; the same investigator assigned patients, gave ESWT and collected all data. Intervention patients had four extra clinic visits, which the authors note could have led to non-comparable wound care, but no deviations are documented. The analysis excluded the two patients lost to follow-up (labeled ITT); with 1 per arm this is unlikely to have a substantial impact.	*"This study was as an open-label randomised clinical…"* *"…it is possible that the groups received non-comparable…"* *"…four extra visits to the clinic for ESWT treatments…"* *"…intention-to-treat principle; patients lost to follow-up…"*
D3 Missing outcome data	**Some concerns**	Outcome data for 21/23 (91%); one patient per arm was lost to follow-up. In the ESWT arm the reason was vascular surgery (suggesting deterioration of perfusion/ulcer), in the control arm unknown. With 10-11 patients per arm a single missing value could shift the mean area reduction; no sensitivity analysis. Missingness could depend on the true outcome, although it is balanced in number.	*"ESWT treatments. In each group, one patient was lost…"* *"…two patients (one in each group) were lost to follow-up;"* *"…due to the small study population, this could have…"*
D4 Measurement of the outcome	**Some concerns**	Ulcer area was traced on digital photographs in ImageJ (three tracings averaged) by the unblinded investigator who also delivered ESWT; the authors acknowledge this could bias the main outcome toward better healing in the intervention group. Tracing borders involves some judgment; judged Some concerns rather than High because a standardized planimetric method was used (borderline High).	*"…particular, measurements of ulcer area were not blinded…"* *"…since the same investigator performing ESWT treatments…"* *"…also measured ulcer area. This could bias the main…"* *"Ulcer area was determined by tracing ulcer borders on…"*
D5 Selection of the reported result	**Low**	Registered at ClinicalTrials.gov (NCT02251418; registered before recruitment started in March 2015 judging by the number); ulcer area reduction at 7 weeks is named as the primary outcome and results at all planned visits (3, 5, 7 weeks) are reported, including the non-significant between-group result. No sign of selective reporting.	*"…at clinicaltrials.gov with registration number…"* *"…primary outcome (ulcer area reduction in the…"* *"…stopped at the pre-specified project duration."*
**Overall**	**Some concerns**	Some concerns in D2, D3, D4 and no domain at high risk, so the overall judgment is Some concerns (standard RoB 2 algorithm; no escalation for multiple Some concerns).	

Dolibog et al., 2018a [106] — RoB 2

Design as reported: Randomized, controlled clinical pilot study, three arms (rESWT vs fESWT vs standard wound care). Design as assessed: RCT (parallel, three-arm). Comparison: rESWT + SWC (group A, n=17) and fESWT + SWC (group B, n=15) vs SWC alone (group C, n=18). Outcome: relative percentage change in ulcer surface area at 8 weeks (4 weeks after the 6th session; primary area outcome). Complete healing was not used because SWC events are reported only graphically (Figure 3), not as numbers.. Participants: 57 randomized; 50 analyzed (17/15/18); 7 did not complete treatment and were excluded (distribution by arm not given in the text).

Domain	Judgment	Rationale	Supporting quotations
D1 Randomization process	**Some concerns**	Random sequence generated with random.org (adequate), but no information on allocation concealment (the 'technician' statement concerns coding of data, not concealment). Baseline characteristics did not differ significantly; mean ulcer area was smaller in the rESWT arm (5.8 vs 8.1 vs 8.3 cm2) but this is compatible with chance.	*"…groups at using website random.org to true random…"*<br>*"Distribution characteristics of the patients did…"*<br>*"…not differ significantly between the groups A, B and…"*
D2 Deviations from intended interventions	**Some concerns**	Open trial without sham: participants and the treating physiotherapist were aware of allocation; no information on deviations arising from the trial context or on co-interventions. The analysis included only those who completed treatment (not ITT); because only 7/57 were excluded, the impact of this is judged mainly under D3.	*"Treatments were performed by an experienced…"*<br>*"Seven patients incomplete treatment (Figure 1)."*
D3 Missing outcome data	**High**	Outcome data are missing for 7/57 randomized participants (12%) who did not finish treatment; their arm and reasons are not reported in the text and no sensitivity analysis is provided. Non-completion of wound therapy plausibly relates to ulcer course (worsening, infection, hospitalization), so missingness could depend on the true value and, with no information on reasons, the RoB 2 algorithm leads to High risk.	*"Patients selected for the treatment (n = 57) were…"*<br>*"…the study without giving a reason, and 7 patients did…"*
D4 Measurement of the outcome	**Some concerns**	Ulcer area was measured by tracing on transparent film and digitizer planimetry (appropriate, same in all groups), but there is no statement that the person tracing/measuring was blinded; tracing ulcer margins involves some judgment, so assessment could have been influenced by knowledge of allocation, though this is not considered likely to be substantial.	*"…subjective based on the examination of the various…"*<br>*"Planimetry was an objective…"*
D5 Selection of the reported result	**Some concerns**	No registration or pre-specified analysis plan. Several area-type outcomes (area, circumference, length, width, Gilman index, nonlinear T1/2) at two time points are reported; the 'primary endpoints' are within-group changes. The between-group area result at 8 weeks is reported in full, but the abstract claims significant superiority of fESWT over SWC, which Table 3 does not show (p(B C)=0.084) and which rests only on the nonlinear T1/2 analysis, indicating selective emphasis.	*"…study endpoints were analysis of changes of the total…"*<br>*"…ulcer surface area and linear dimensions inside…"*<br>*"There is statistically significant difference between the standard care and radial shockwave therapy…"*
**Overall**	**High**	High because at least one domain is High (D3); some concerns also in D1, D2, D4, D5 (standard RoB 2 algorithm).	

Dolibog et al., 2018b [112] — RoB 2

Design as reported: Randomized clinical pilot study, three arms (ultrasound therapy vs rESWT vs standard care), 1:1:1. Design as assessed: RCT (parallel, three-arm). Comparison: rESWT + SWC (group B, n=17) vs SWC alone (group C, n=17); ultrasound arm (group A) not considered. Outcome: mean percentage reduction in ulcer area at 4 weeks (primary; no complete-healing data reported). Participants: 51 randomized (17/17/17); 51 analyzed (all completed).

Domain	Judgment	Rationale	Supporting quotations
D1 Randomization process	**Some concerns**	Patients were 'randomly assigned' but the sequence generation method and allocation concealment are not described. Groups were declared homogeneous, yet body weight differed significantly ($p<0.05$) and SWC ulcers were larger (median 11.2 vs 4.6 cm2); these imbalances may be chance in a small trial but together with no concealment information they give some concerns.	*"…and randomly assigned to three comparison groups in…"* *"Patients in all comparison groups were homogeneous…"*
D2 Deviations from intended interventions	**Some concerns**	No sham or blinding; participants and physiotherapists knew the allocation. No information on deviations from intended interventions; all participants completed and were analyzed in their groups.	*"…sound and shock wave therapy) and nurses (standard…"*
D3 Missing outcome data	**Low**	All 51 randomized participants completed the study and were analyzed.	*"All participants completed the entire study pro-…"*
D4 Measurement of the outcome	**Some concerns**	Ulcer area was measured by digital planimetry (appropriate, same in all groups) by nurses who were part of the treating team; no blinding of outcome assessors is reported. Tracing involves some judgment, but strong influence is not considered likely.	*"…wound area measurements, data collections)."* *"…using digital planimetry (Kurta XGT, Altek Inc,…"*
D5 Selection of the reported result	**Some concerns**	No registration, protocol or pre-specified analysis plan is cited. Area change and Gilman index at 4 weeks are both reported; no clear evidence of selection, but pre-specification cannot be verified.	—
**Overall**	**Some concerns**	Some concerns in D1, D2, D4, D5 and no domain at high risk, so the overall judgment is Some concerns (standard RoB 2 algorithm; no escalation for multiple Some concerns).	

Snyder et al., 2018 [28]* — RoB 2

Design as reported: Two sequential multicenter, prospective, randomized, double-blind, parallel-group, sham-controlled phase III trials (Study 1 NCT00536744, 2007-2010; Study 2 NCT01824407, 2013-2015) plus a post hoc pooled analysis. Design as assessed: RCT (parallel) x 2; pooled (simple patient-level pooling) analysis of two RCTs. Comparison: focused electrohydraulic ESWT (dermaPACE) + standard care vs sham ESWT + standard care. Outcome: complete wound closure at 24 weeks (patients; pooled Study 1 + Study 2: 65/172 vs 43/164, all randomized in denominator). Participants: 336 randomized (Study 1: 107 vs 99; Study 2: 65 vs 65); pooled analysis 172 vs 164 (all randomized); completed 24 weeks 122 vs 113 (Study 1: 82 vs 74; Study 2: 40 vs 39).

Domain	Judgment	Rationale	Supporting quotations
D1 Randomization process	**Low**	Study 1: allocation by sealed envelopes prepared before the study, stratified by diabetes type, opened sequentially from a locked box and held by unblinded application administrators (sequence-generation method not described; envelopes not stated to be opaque). Study 2: randomly permuted blocks stratified by site and ulcer duration; concealment not separately described but design stated to be 'largely identical' to Study 1. Baseline differences (Study 1: older age and longer diabetes duration in the active arm; pooled: older ulcers and more type 1 DM in the sham arm) are in mixed directions and compatible with chance. Concealment judged probably adequate in both trials, hence Low (borderline Some concerns, see notes).	*"Randomisation was stratified by diabetes type (type 1…"*<br>*"…by sealed envelopes prepared before the study started…"*<br>*"…envelopes were opened sequentially after removal from…"*<br>*"Randomisation was balanced by using randomly…"*<br>*"…permuted blocks stratified by study site and target ulcer…"*<br>*"However, the mean age of subjects and duration…"*
D2 Deviations from intended interventions	**Low**	Participants, evaluating investigators, planimetry staff and coordinators were blinded; a dummy applicator with sound simulation was used for sham; only the application operator was unblinded and the PI was excluded from the treatment room. No deviations from the protocol were reported (Galiano et al., 2019 [90]). The pooled 24-week analysis used all randomized patients (ITT = mITT because every randomized patient received at least one application).	*"…by an unblinded operator. The principal investigator…"*<br>*"…planimetry, and study coordinators were all blinded to…"*<br>*"In both studies the ITT population was defined as all…"*<br>*"…the ITT and MITT populations was the same because all…"*
D3 Missing outcome data	**Some concerns**	Only 122/172 (71%) active and 113/164 (69%) sham patients completed 24 weeks (Study 1: 82/107 vs 74/99; Study 2: 40/65 vs 39/65). The 24-week proportions use all randomized patients as denominators, i.e. withdrawals are effectively counted as not healed; no sensitivity analysis for this assumption is reported. Withdrawals were partly due to adverse events, so missingness could depend on the healing status; however, the proportions and reported reasons are similar between arms, so bias is possible but not clearly likely.	*"…calculated using all enrolled subjects…"*<br>*"…were randomised, 113 remained enrolled in the trial at…"*<br>*"…the 12-week time point, and 79 remained at the 24-week…"*<br>*"There were 42 subjects who withdrew in the…"*
D4 Measurement of the outcome	**Low**	Complete closure (re-epithelialization without drainage or dressing, confirmed at two consecutive visits) was determined by blinded evaluating investigators using serial photography and tracings (central imaging laboratory in Study 1; Aranz SilhouetteStar plus an external third-party photo adjudication in Study 2). Method identical between arms.	*"Wound closure, determined by the blinded evaluating…"*<br>*"…closure was determined both by the investigator and an…"*<br>*"…external third party that assessed photographs."*
D5 Selection of the reported result	**Some concerns**	Both trials were registered and complete closure was the pre-specified primary outcome, but at 12 weeks; 24 weeks was a pre-planned follow-up time point. The pooled analysis on which the review relies was not pre-specified: it was undertaken after Study 2 'did not produce outcomes in line with expectations' to support an FDA de novo submission. Study 2's pre-specified primary analysis (Bayesian design; Cox HR in the mITT population) is not reported numerically. Because the per-trial results at 12, 20 and 24 weeks are all reported in Table 4, the pooled estimate can be reproduced from fully reported trial-level data, which limits (but does not remove) the concern.	*"Study 1 (ClinicalTrials.gov Identifier: NCT00536744):"*<br>*"Study 2 (ClinicalTrials.gov Identifier: NCT01824407)"*<br>*"…incidence of complete wound closure within 12 weeks."*<br>*"Study 2 did not produce outcomes in line with expectations…"*<br>*"…required to satisfy the requirements for a de novo pathway…"*

Domain	Judgment	Rationale	Supporting quotations
**Overall**	**Some concerns**	Some concerns in D3, D5 and no domain at high risk, so the overall judgment is Some concerns (standard RoB 2 algorithm; no escalation for multiple Some concerns).	

Taheri et al., 2021 [102] — RoB 2

Design as reported: Double-blind parallel-group randomized clinical trial, multicenter (Isfahan, Iran, 2018-2019); IRCT20190908044730N2. Design as assessed: RCT (parallel). Comparison: focused electromagnetic ESWT (4 weekly sessions) + compression bandaging vs sham ESWT (device switched off) + compression bandaging. Outcome: wound size at week 8 (primary wound outcome; complete healing not reported): 94.8 +/- 176.2 vs 307.6 +/- 433.6 (units stated cm2), time x group p=0.281. Participants: 50 randomized (25 vs 25); 44 analyzed (22 vs 22).

Domain	Judgment	Rationale	Supporting quotations
D1 Randomization process	**Some concerns**	Sequence generated with Random Allocation Software using block randomization (adequate), but allocation concealment is not described. Baseline characteristics are given only for the 44 analyzed patients; baseline wound size is larger in the control group (715.8 vs 567.8) with very large SDs, and the control-group wound-duration SD (3.6 weeks) is implausible. No clear evidence of a randomization problem, but concealment is unknown.	*"…ceiving sham ESWT) using random allocation…"*<br>*"…software version 2 and block randomization…"*<br>*"Wound duration (week)"*
D2 Deviations from intended interventions	**Some concerns**	Sham with the device switched off; described as double-blind, but ESWT-induced pain is reported, so participants may have recognized active treatment, and the operator was presumably aware. No information on deviations. Analysis was restricted to completers (44/50; 3 per arm excluded for being 'reluctant to continue'), i.e. not ITT; with 12% balanced exclusions the impact is probably not substantial.	*"The current double-blind parallel-group ran-…"*<br>*"…the waves (the device was turned off for this…"*<br>*"WT-induced pain, which initially caused pa-…"*<br>*"…continue cooperation. The final analysis was…"*
D3 Missing outcome data	**Some concerns**	Outcome data available for 44/50 (88%), 3 missing per arm, all described only as unwilling to continue cooperation. Non-cooperation could relate to pain or poor healing, so missingness could depend on the true outcome; no sensitivity analysis, but numbers are balanced.	*"…tients in the ESWT group and three patients…"*<br>*"…study during follow-up and were reluctant to…"*<br>*"…performed on 22 patients in the ESWT group…"*
D4 Measurement of the outcome	**Some concerns**	The method of wound-size measurement is not described and the authors acknowledge possible measurement error; it is unclear whether the wound assessor was blinded ('the analyzer was also unaware' may refer to the data analyst). Participant blinding may have been compromised (perceptible/painful ESWT vs switched-off device). Wound size is a measured outcome, so a strong influence is not likely.	*"The analyzer was also unaware of the blinding…"*<br>*"…bility of error in measuring the ulcer size."*
D5 Selection of the reported result	**Some concerns**	Registered in IRCT, but the registration number (dated 2019-09-08) suggests registration during or after the 2018-2019 study period, and no protocol or analysis plan is cited. Several outcomes (wound size, pain, satisfaction, CCVUQ subscales, drainage) were measured at two time points; wound-size results are fully reported (non-significant), so no evidence of selection of a favorable wound-size result, but pre-specification cannot be confirmed. The abstract misreports the wound-size and pain findings as significant.	*"IRCT (IRCT20190908044730N2)."*<br>*"The significant efficiency of ESWT in im-…"*<br>*"…and ulcer size in the two groups showed no…"*<br>*"…significant difference (P>0.05)."*
**Overall**	**Some concerns**	Some concerns in D1, D2, D3, D4, D5 and no domain at high risk, so the overall judgment is Some concerns (standard RoB 2 algorithm; no escalation for multiple Some concerns).	

Fan et al., 2022 [25] — ROBINS-I V2

Design as reported: Not explicitly stated. The full text never uses the words 'random', 'randomized' or 'allocation'; it describes 87 admitted patients 'enrolled in this study for analysis', of whom 46 received ESWT 'while the rest 41' received routine treatment. (The review's file name and the Supplementary File 5 summary sentence call it an RCT.). Design as assessed: cohort with concurrent controls (probably retrospective, based on hospital records). Comparison: Radial ESWT (Swiss DolorClast Smart, weekly for 4–6 weeks) + routine treatment and nursing intervention vs routine treatment and nursing intervention. Outcome: Wound reduction rate (%) after the treatment period (4–6 weeks), the study's primary wound outcome (healing time is also reported). Complete healing is not reported as events; results appear only in Figure 1 with P<0.05, without numerical values.. Participants: 87 included (46 ESWT, 41 control); the number analyzed is not stated (Table 2 percentages imply 44 in the ESWT group).

Domain	Judgment	Rationale	Supporting quotations
D1 Confounding	**Serious**	Nothing is said about how patients came to receive ESWT or not. There is no randomization, matching or adjusted analysis. The measured baseline variables (age, sex, BMI, disease course, ulcer area, lifestyle) are similar, but key prognostic factors (ulcer grade/depth, infection severity, glycemic control, perfusion/PAD, ulcer duration) are neither reported nor controlled for.	*"Among them, 46 patients received ESWT…"*<br>*"…combined with nursing intervention as the research…"*<br>*"…admitted between March 2018 and May 2020 were…"*
D2 Classification of interventions	**Moderate**	Receipt of ESWT is probably clear from the records. However, the course length varied (4–6 weeks), possibly according to response, and with apparent retrospective classification it is not certain that intervention status was defined at baseline independently of outcome.	*"The shock therapy was carried out for…"*<br>*"…each patient 1 time/week for 4-6 weeks in total."*
D3 Selection of participants into the study (or into the analysis)	**Moderate**	Patients were included only if they had 'detailed clinical data', which may exclude patients who left treatment early or deteriorated; the start of follow-up relative to the start of ESWT is not described. Whether this selection is related to both intervention and outcome cannot be determined.	*"Patients with detailed clinical data, and those…"*<br>*"…admitted between March 2018 and May 2020 were…"*
D4 Deviations from intended interventions	**Moderate**	Both groups received the same background treatment (hypoglycemics, anti-infectives, alprostadil, lipoic acid, daily debridement, nursing). Patients and staff were aware of the intervention, and no information is given on adherence or on differential co-interventions; the description of the control group's wound disinfection is unclear.	*"Both groups were given routine hypoglycemic…"*<br>*"…injection of alprostadil injection and lipoic acid for…"*
D5 Missing data	**Moderate**	No information on missing outcome data or on the number analyzed. The Table 2 percentages for the ESWT group (2/4.55%, 42/95.45%) imply a denominator of 44, not 46, suggesting that some data are missing without explanation.	*"…the control group (95.45% vs. 80.49%, P<0.05)."*<br>*"2(4.55)"*
D6 Measurement of the outcome	**Moderate**	The method of measuring wound area and calculating the reduction rate is not described. Assessors were presumably aware of the intervention received, so assessment could have been influenced, although wound area is a relatively objective measure.	*"The reduction rate and healing time of wound…"*<br>*"As shown in Figure 1, the wound reduction rate…"*
D7 Selection of the reported result	**Moderate**	There is no protocol or registration. Many outcomes are reported, most only as figures with 'P<0.05' and no numerical estimates, so selection among analyses or measurements cannot be excluded.	*"…(%) of the research group was greatly higher than…"*
**Overall**	**Serious**	Serious risk of bias because of uncontrolled confounding in a non-randomized comparison (D1), with moderate concerns in all other domains.	

Dymarek et al., 2023 [115] — RoB 2

Design as reported: Preliminary, prospective, double-blinded randomized controlled trial (active vs sham radial ESWT, single session), part of the SHOWN project. Design as assessed: RCT (parallel). Comparison: single rESWT session + SWC vs sham rESWT + SWC. Outcome: change in planimetric wound area from baseline (M0) to 1 week after a single session (M1); no complete-healing data. WBS and BWAT are additional clinical scores.. Participants: 40 randomized (20/20); 40 analyzed.

Domain	Judgment	Rationale	Supporting quotations
D1 Randomization process	**High**	Sequence generated by a website randomization tool (adequate), but allocation concealment is not described. Baseline wound-condition scores differed markedly between groups (WBS 3.85 vs 7.10, p=0.006; BWAT 45.45 vs 34.05, p=0.0004; active group much worse), contradicting the authors' statement of homogeneous baseline characteristics. With no concealment information and baseline imbalance in prognostic wound status, RoB 2 leads to High.	*"…a website-generated randomization tool. All pa-…"*<br>*"…baseline characteristics (p > 0.05)."*<br>*"…score from 3.85 to 9.65 points…"*
D2 Deviations from intended interventions	**Low**	Patients were blinded with a sham transducer cover; only the treating physiotherapist knew the allocation. Single-session intervention, standard care continued in both groups, all randomized patients analyzed in their groups.	*"…tients were blinded to their radial ESWT inter-…"*<br>*"…vention, and only the researcher performing the…"*<br>*"…study and were analyzed."*
D3 Missing outcome data	**Low**	All 40 randomized patients completed the study and were analyzed. Two deaths 'during the study' are listed among pre-randomization exclusions; if they occurred after randomization, 2/42 would be missing, which is unlikely to bias the result materially.	*"…during the study (n = 2). Finally, 40 patients were…"*
D4 Measurement of the outcome	**Low**	Wound area measured with a validated non-contact smartphone planimetry application (objective, same in both groups); the report states only the treating physiotherapist knew the allocation, implying blinded assessment. It is not explicitly stated who performed the measurements.	*"…smartphone application Swift Skin and Wound…"*<br>*"…vention, and only the researcher performing the…"*
D5 Selection of the reported result	**Some concerns**	The SHOWN project was prospectively registered (ACTRN12617000075381), but the paper is a 'preliminary' report of a single-session, 1-week analysis from a broader project (study period 2017-2021, other SHOWN publications exist), and no pre-specified analysis plan for this report is described; consistency of the outcome/time point with the registry could not be verified from the full text. All measured outcomes (area, length, width, WBS, BWAT) are reported.	*"Platform (No. ACTRN12617000075381)."*<br>*"…and 1 week after radial ESWT sessions…"*
**Overall**	**High**	High because at least one domain is High (D1); some concerns also in D5 (standard RoB 2 algorithm).	

Jeong et al., 2023 [92] — ROBINS-I V2

Design as reported: Prospective (observational) study; ulcerated feet = ESWT group, unaffected contralateral feet = control group. Design as assessed: non-randomized within-patient comparison (ESWT-treated ulcerated feet vs untreated non-ulcerated contralateral feet); in effect a before-after study with a non-comparable reference group. Comparison: fESWT (orthoPACE, 3x/week for 3 weeks) + standard dressing/debridement on ulcerated feet vs no treatment on non-ulcerated contralateral feet. Outcome: No wound healing/closure or wound area outcome reported. Primary outcome: $TcPO_2$ (mm Hg, dorsal foot) at week 3 (also weeks 1 and 2), between groups and vs baseline. Participants: 25 patients; 50 feet (32 ulcerated ESWT feet, 18 non-ulcerated control feet); all analyzed.

Domain	Judgment	Rationale	Supporting quotations
D1 Bias due to confounding	**Critical**	Group membership is the disease state itself: ulcerated feet were treated and healthy contralateral feet were controls. The groups therefore differ by definition in the main determinant of the outcome, and baseline $TcPO_2$ was 34.3 vs 52.9 mm Hg. No analysis can separate the ESWT effect from the ulcer/no-ulcer difference, and the authors themselves call for careful interpretation.	*"The affected feet were used as the ESWT group and the unaffected contralateral feet…"*<br>*"…pared to the baseline (baseline: 34.34 ± 3.92 mm Hg vs 1st…"*<br>*"52.89 ± 3.85 mm Hg vs 1st measurement…"*<br>*"…between the ESWT and control groups, careful interpretation…"*
D2 Bias in classification of interventions	**Low**	Treatment status was set by ulcer status at baseline and is unambiguous.	*"…ulcerated feet comprised the ESWT group, while the contralat- …"*
D3 Bias in selection of participants into the study (or into the analysis)	**Low**	Patients meeting the stated criteria were recruited prospectively, and follow-up started with the intervention. Selection of control feet (contralateral feet without ulcer; 7 patients with bilateral ulcers contributed no control) is part of the confounding problem rather than selection based on post-intervention events.	*"25 type 2 diabetes mellitus patients (19…"*
D4 Bias due to deviations from intended interventions	**Moderate**	Open study. All ESWT feet reportedly received 9 sessions. Standard dressing and debridement, which may affect local perfusion, went only to the ESWT (ulcerated) feet. Deviations are not otherwise reported.	*"…sessions over the course of 3 weeks…"*<br>*"…patients underwent adjunctive standard dress-…"*
D5 Bias due to missing data	**Low**	Figures report all 32 ESWT and 18 control feet at every time point, and no losses are reported. The CONSORT diagram is not available in the text file.	*"…(n = 32 for the ESWT…"*<br>*"…group and n = 18 for the control group)"*
D6 Bias in measurement of the outcome	**Low**	$TcPO_2$ was measured with a device under a standardized protocol (fixed site, electrode temperature, room temperature) in both groups. Assessors were not blinded, but an instrument reading is unlikely to be influenced materially.	*"$TcPO_2$ (mm Hg) was measured using a TCM 400 device…"*<br>*"…attached to the skin of the dorsal part of the foot, in the first…"*
D7 Bias in selection of the reported result	**Moderate**	A trial registration is mentioned in the eligibility criteria, but no registry ID or pre-specified analysis plan is given. Results are reported for all time points and subgroups, but the analysis description (ANOVA, paired and independent t tests, mean ± SEM) does not match the presentation, and between-group differences are given as '%p'.	*"…extracellular matrix) within 30 d of the trial registration…"*<br>*"One-way analysis of variance was used to compare the sig-…"*
**Overall**	**Critical**	D1 is Critical: treated ulcerated feet are compared with untreated healthy feet, so the between-group comparison cannot estimate an effect of ESWT, and the overall judgment takes the worst domain.	

Vangaveti et al., 2023 [119] — RoB 2

Design as reported: Prospective, single-center, randomized clinical trial, parallel, two arms (unblinded). Design as assessed: RCT (parallel). Comparison: ESWT every 2 weeks + standard of care vs standard of care alone. Outcome: Complete healing of the index ulcer at 6 weeks (patients): 5/25 ESWT + SOC vs 6/23 SOC. Participants: 48 randomized (25 ESWT + SOC, 23 SOC); 9 lost to follow-up (5 vs 4); complete healing reported against the randomized denominators (48).

Domain	Judgment	Rationale	Supporting quotations
D1 Randomization process	**Low**	A web-based randomization program (Research Randomizer) generated the sequence, and allocation used sealed envelopes accessible only to the research nurse; the envelopes are not described as opaque or sequentially numbered. Baseline characteristics were broadly similar; the few differences (e.g. ATSI, hyperlipidemia, IL-1β) are compatible with chance.	*"…using a web based online program (20) in a 1:1 ratio…"*<br>*"…was then placed in sealed envelopes with research nurse being the only person having…"*<br>*"…the characteristics were similar in both groups."*
D2 Deviations from intended interventions	**Some concerns**	The trial was open (no sham), and the authors acknowledge possible investigator bias and variation in offloading materials. Nothing is reported on deviations from intended interventions. Missing data were excluded from analyses; the complete-healing counts use randomized denominators, but it is unclear how the patients lost to follow-up were counted.	*"The treatment group was not blinded and could have led to…"*<br>*"Missing data were excluded…"*
D3 Missing outcome data	**Some concerns**	9 of 48 randomized patients (19%; 5 ESWT, 4 SOC) were lost to follow-up, a number similar to the total of 11 healing events. Reasons are not given in the text, and complete healing is reported over 48, which implies the lost patients were counted as unhealed or that their status was otherwise known; this is not explained. Missingness in DFU trials (e.g. hospitalization, amputation) can relate to the true outcome. Proportions were similar across arms, so 'Some concerns' rather than 'High'; the authors should check the flow diagram.	*"…the SOC group and 5 (22%) in the ESWT + SOC group were lost to…"*<br>*"…(26.1%) in the SOC group and 5 out of 25 in the (20.0%) in the ESWT…"*
D4 Measurement of the outcome	**Some concerns**	Wounds were measured with a 3D camera, using the same method in both arms, but by the same unblinded team that delivered treatment. A judgment of complete healing by an unblinded assessor could be influenced by knowledge of assignment, although the 3D measurement limits this.	*"3D wound measurement camera, Aranz medical, Christchurch,…"*<br>*"…clinical measurements and treatments, eliminating the possible inter-…"*<br>*"The treatment group was not blinded and could have led to…"*
D5 Selection of the reported result	**Some concerns**	A study protocol is mentioned but no trial registration is cited. The primary outcome ('change in wound healing') was analyzed in several ways: a dichotomy at ≥20% area change with logistic regression, complete healing, and percentage area reduction. The abstract reports ORs (3.2, p = .07; 3.9, p = .08) that differ from those in the Results and Table 4 (2.5, p = .23; 2.9, p = .26), so it is unclear which analysis was pre-specified.	*"This study was performed following the protocol…"*<br>*"…(OR = 3.2, p = .07)"*<br>*"…weeks with an unadjusted OR of 2.5 (0.54-1.21), p = .0.23 (Table 4)"*<br>*"…based on a minimum of 20% change between the wound surface area at…"*
**Overall**	**Some concerns**	Some concerns in D2, D3, D4, D5 and no domain at high risk, so the overall judgment is Some concerns (standard RoB 2 algorithm; no escalation for multiple Some concerns).	

Dolibog et al., 2024 [24] — ROBINS-I V2

Design as reported: Single-center 'randomized' study, four arms (IPC vs rESWT vs fESWT vs standard care), reported per CONSORT. Design as assessed: non-randomized controlled study with sequential (phase-wise, non-concurrent) allocation by order of presentation; standard-care controls recruited last. Comparison: rESWT + SC (n=16) and fESWT + SC (n=15) vs SC alone (n=18); IPC arm (n=20) not considered. Outcome: relative percentage change in ulcer surface area (RCA) at 4 weeks (primary). Complete healing occurred only in the IPC arm (2 patients), i.e. no events in the ESWT and SC arms, so the primary area outcome was assessed.. Participants: 69 included (IPC 20, rESWT 16, fESWT 15, SC 18); number screened/excluded or lost not reported in the text.

Domain	Judgment	Rationale	Supporting quotations
D1 Bias due to confounding	**Serious**	Although called 'randomized', patients were assigned by recruitment phase (IPC first, then rESWT, then fESWT, then SC), so groups come from different calendar periods over 5 years with no adjustment for confounders. Baseline differences are present (fESWT 67% male vs SC 17%; ulcer duration median 12/10 vs 18.5 months; area 7.4/6.1 vs 10.5 cm2) and secular changes in care/referral cannot be excluded. Allocation was not by clinician choice, so Serious rather than Critical.	*"…were recruited for treatment using intermittent pneumatic compression therapy. Upon…"*<br>*"…completion of this phase, patients were recruited to the radial extracorporeal shockwave…"*<br>*"…patients were assigned to a treatment group, there could be some bias to the results."*
D2 Bias in classification of interventions	**Low**	Intervention groups were defined at the start of treatment by recruitment phase and the protocols (device, sessions, parameters) are clearly specified; classification is not affected by outcome knowledge.	*"…therapy group. Subsequently, the focus shifted to focal extracorporeal shockwave thera-…"*
D3 Bias in selection of participants into the study (or into the analysis)	**Moderate**	Start of follow-up coincides with start of treatment, but patients were enrolled knowing which single treatment was being offered in each phase, so willingness to participate may differ by phase; numbers screened, excluded or not analyzed are not reported in the text (CONSORT figure only), so selection into the analysis cannot be checked.	*"The final study included 69 patients whose characteristics were assessed before random-…"*
D4 Bias due to deviations from intended interventions	**Moderate**	Unblinded interventions delivered by physiotherapists and nurses; no information on adherence, missed sessions or co-interventions; standard care was the same daily regimen in all groups. No evidence of important deviations, but none reported.	*"All patients in all groups were treated by dermatologists (treatment coordination),…"*
D5 Bias due to missing data	**No information**	The text does not report how many allocated patients dropped out or were excluded from the analysis ('final study included 69'); the companion 2018 trial from the same protocol excluded 12% non-completers. Missing-data handling cannot be assessed from the text.	—
D6 Bias in measurement of the outcome	**Moderate**	Ulcer area measured by non-contact digital planimetry from photographs (objective, high accuracy, same method in all groups), but assessors were not stated to be blinded and the wound-margin delineation involves some judgment.	*"The wound healing rates were assessed on the basis of using high-resolution digital…"*<br>*"…photographs with markers and non-contact digital planimetry."*
D7 Bias in selection of the reported result	**Moderate**	No registration/protocol cited. The stated primary endpoint was relative change in ulcer area AND perimeter at 4 weeks, but perimeter results are not reported; pairwise RCA comparisons for the ESWT arms vs SC are given only as effect-size categories without p-values, while overall Kruskal-Wallis p=0.001 is driven by IPC.	*"The primary endpoint of the study outcome was wound relative change in the ulcer…"*<br>*"…surface area and perimeter after 4 weeks of treatment."*
**Overall**	**Serious**	Serious risk of confounding from sequential, non-concurrent phase allocation without adjustment (most severe domain judgment).	

Hitchman et al., 2025 [27] — RoB 2

Design as reported: Single-center pilot three-arm double-blinded randomized controlled trial (high-dose ESWT vs low-dose ESWT vs sham ESWT, all plus standard DFU care). Design as assessed: RCT (parallel, three-arm). Comparison: fESWT + SWC (high-dose arm 500 shocks/cm2 and low-dose arm 100 shocks/cm2, pooled) vs sham ESWT + SWC. Outcome: complete healing at 24 weeks (patients); 16/25 high dose, 13/23 low dose, 11/26 sham (pooled active arms 29/48 vs 11/26). Participants: 74 randomized (25 high dose, 23 low dose, 26 sham); 74 analyzed (ITT); 7 lost to follow-up (3/3/1) and 1 discontinued after healing.

Domain	Judgment	Rationale	Supporting quotations
D1 Randomization process	**Low**	Stratified block randomization with randomly varying block sizes via an online tool developed by an independent trials-unit statistician; the research team had no access to the allocation sequence (adequate concealment). Baseline differences (age, ulcer area, ulcer duration, SWHSI) are noted by the authors; they are compatible with chance in a small trial and, for ulcer area, favor the sham arm, so they do not suggest a problem with the randomization process.	*"Stratified block randomisation, using randomly varying block…"*<br>*"…was developed by an independent statistician at York Trials…"*<br>*"The research team did not have access to the allocation…"*
D2 Deviations from intended interventions	**Low**	Participants were blinded by a sham procedure (machine prepared identically, audio recording played); all 74 received their allocated intervention (4 missed single sessions, balanced) and all were analyzed in their randomized arms by intention-to-treat. Personnel delivering ESWT were not stated to be blinded, but no deviations arising from the trial context are reported.	*"Trial participants were blinded to the treatment allocation."*<br>*"…was played. Participants received the intervention three times…"*<br>*"Analyzed (n = 25)"*
D3 Missing outcome data	**Some concerns**	Healing status at 24 weeks was not observed for 7/74 (9.5%) participants lost to follow-up (3 high dose, 3 low dose, 1 sham); the paper does not state how they were handled in the healed proportions (denominators are the full randomized numbers, implying they were counted as unhealed). Missingness was higher in the active arms and attendance may be related to healing status (authors suggest healed patients disengage), so it could depend on the true outcome; no sensitivity analysis is reported. The likely direction would underestimate the ESWT effect, but bias cannot be excluded.	*"…(DFU healed, did not want…"*<br>*"…creased over time, with a greater reduction in the active ESWT…"*<br>*"…only 12.2%, significantly less than the expected 30%."*
D4 Measurement of the outcome	**Low**	Healing had a clear definition (complete epithelialization without eschar sustained for 2 weeks) and was confirmed by the research team; clinicians assessing healing were blinded to arm and participants were blinded via sham, so assessment is unlikely to have been influenced by knowledge of allocation.	*"Clinicians assessing whether the DFU was healed were blinded…"*<br>*"Healing was reported by the patient or a clinician."*<br>*"The patient was then reviewed by the research team within…"*
D5 Selection of the reported result	**Some concerns**	No trial registration number or published protocol/statistical analysis plan is cited in the paper (ethics approval only). Healing was a pre-specified secondary outcome and is reported at all follow-up points (6, 12, 24 weeks) for all arms, descriptively without testing, so there is no specific signal of selective reporting; but pre-specification cannot be verified from the paper.	*"Statistical testing was not performed as this is a pilot RCT…"*<br>*"24 weeks, 64.0% (16/25) of DFUs healed in the high dose ESWT…"*
**Overall**	**Some concerns**	Some concerns in D3, D5 and no domain at high risk, so the overall judgment is Some concerns (standard RoB 2 algorithm; no escalation for multiple Some concerns).	

Ishii et al., 2025 [104] — ROBINS-I V2

Design as reported: Phase 3 pivotal, non-randomized, parallel-group trial with conventional-treatment controls (UMIN000012349). Design as assessed: cohort with concurrent controls (non-randomized controlled trial; ESWT at 2 centers, controls at 12 other centers). Comparison: fESWT (Duolith SD1, weekly x 8) + continued prior treatment vs conventional treatment (all available therapies). Outcome: Complete healing not reported as events per group; primary outcome assessed: mean reduction in number of digital ulcers at 8 weeks (ESWT 4.47 ± 2.65 vs conventional 0.83 ± 2.79; n = 30 vs 30). Participants: 60 patients (30 ESWT, 30 conventional); 60 in the primary analysis; 49 (21 vs 28) in the propensity-score-adjusted analysis.

Domain	Judgment	Rationale	Supporting quotations
D1 Bias due to confounding	**Serious**	Allocation was by center: ESWT at two specialized facilities, controls at 12 others. The authors say more severe and refractory cases were selected into the ESWT group. Baseline ulcer count, which directly drives the outcome (absolute reduction), was much higher with ESWT (5.57 vs 3.0), and background vasoactive therapy differed. The primary analysis is unadjusted. A propensity-score-adjusted analysis (including baseline ulcer count) was reported as supportive, but it was complete-case (21/28 patients) and could not address confounding by center.	*"The study design was a non-randomised, parallel-group trial…"*<br>*"ESWT at two designated facilities specialising exclusively…"*<br>*"…conventional treatment at 12 facilities that provided standard…"*<br>*"…the time of enrolment, the ESWT group had a higher average…"*<br>*"…with severe and refractory cases to be selected in the ESWT…"*<br>*"…squared tests. We conducted an additional analysis using…"*
D2 Bias in classification of interventions	**Low**	Intervention status was defined at enrollment by treating center and protocol and is unambiguous.	*"The treatment regimen consisted of weekly…"*
D3 Bias in selection of participants into the study (or into the analysis)	**Low**	Both groups used the same eligibility criteria, and follow-up started at the start of treatment. There is no evidence of selection based on post-intervention characteristics; differences in case mix are addressed under D1.	*"Eligibility for the study required…"*<br>*"A total of 60 patients were enrolled, with 30 patients allocated…"*
D4 Bias due to deviations from intended interventions	**Moderate**	Open-label. The control group could receive any available therapy while the ESWT group continued its existing treatments, so co-interventions differed (this would, if anything, favor controls) and are not quantified. All participants completed the protocol, but treatment varied across the 12 control centers.	*"The conventional treatment group was allowed access…"*<br>*"…to all currently available therapies, whereas the ESWT group…"*<br>*"…continued with the treatments they received prior to the study."*
D5 Bias due to missing data	**Low**	All 60 participants completed the study and were included in the primary (unadjusted) analysis. The propensity-adjusted analysis included only 21 ESWT and 28 control patients, with no explanation; that would be a concern if the adjusted result were the one assessed.	*"…tional treatment group. All participants completed the study in…"*
D6 Bias in measurement of the outcome	**Serious**	Ulcer counts were made by unblinded investigators, and the authors state that participant and examiner both knew the allocation. Assessment differed systematically between groups: ESWT patients were evaluated weekly by ESWT-center staff, controls every 4 weeks by different physicians at 12 centers. Deciding what counts as an ulcer (loss of epithelialization, excluding fissures) involves judgment, and the authors note poor reliability of ulcer assessment.	*"…fore, both the participant and examiner can recognise when…"*<br>*"Once per week…"*<br>*"Once per 4 weeks…"*<br>*"…of skin ulcers caused by SSc is challenging."*
D7 Bias in selection of the reported result	**Low**	The trial was registered (UMIN000012349). The primary endpoint (mean reduction in ulcer count at 8 weeks) is pre-specified with a sample-size calculation and reported as planned. Secondary endpoints are reported including non-significant ones (12 wk, VAS, HAQ, EQ-5D, mRSS). Consistency with the registry entry was not verified here.	*"Clinical Trials Registry (UMIN ID: UMIN000012349) to…"*<br>*"The primary endpoint of this study was mean reduction…"*
**Overall**	**Serious**	D1 (allocation by center with a large baseline imbalance in ulcer count and an unadjusted primary analysis) and D6 (unblinded, differently timed assessment by different centers) are Serious, so the overall judgment is Serious.	

Landscheidt et al., 2025 [105] — ROBINS-I V2

Design as reported: Prospective randomized, blinded, monocentric within-person clinical trial (split-thickness skin graft donor and recipient sites divided into halves A/B; Group 1 verum vs placebo halves, n=25; Group 2 sham on both halves, n=10). Design as assessed: quasi-randomized within-person (split-wound) controlled study: the half receiving ESWT was allocated by alternation of patient ID; group membership (Group 1 vs 2) by drawing lots. Comparison: focused electromagnetic ESWT (Duolith SD1, Days 5/7/9) on one half vs placebo (device off) on the other half of the same wound, both with standardized wound care. Outcome: re-epithelialization (proportion of wound area epithelialized, planimetry) at Day 12, within-person comparison ESWT half (1A) vs placebo half (1B) at skin-graft recipient (chronic wound) and donor sites; donor 0.92 vs 0.82 ($p<0.05$), recipient residual area smaller ($p=0.008$). Participants: 35 included; 25 in the within-person ESWT vs placebo comparison (Group 1), 10 in the sham-only group (Group 2); 31 completed to Day 90.

Domain	Judgment	Rationale	Supporting quotations
D1 Bias due to confounding	**Moderate**	The within-person comparison removes patient-level confounding. However, which half (upper or lower) received ESWT was allocated by alternation of patient ID, i.e. predictable and not random; the investigator could know in advance which half would be treated when dividing the wound into A/B. Alternation counterbalances upper vs lower position across patients, but no data on the comparability of the two halves (size, location, wound bed) are reported.	*"The allocation to wound Area A and wound Area B took place…"*<br>*"…in alternation. If the upper wound area was treated in the…"*<br>*"…participant with ID 01, then the lower area was treated in the…"*<br>*"Randomization was performed randomly by drawing lots to…"*
D2 Bias in classification of interventions	**Low**	Intervention status of each half (verum A vs placebo B) was defined at the start and applied at fixed visits (Days 5, 7, 9); misclassification is unlikely.	*"…shock waves (verum, Area A), the other with placebo (Area B)."*<br>*"Group 1B = placebo therapy (device off, split skin donor and split…"*
D3 Bias in selection of participants into the study (or into the analysis)	**Low**	Consecutive patients needing split-thickness grafting (May 2016 - July 2020); because both interventions are applied within each participant, selection into the study cannot be related to intervention, and follow-up started at the time of intervention.	*"In the period from May 2016 to July 2020, 35 participants with…"*
D4 Bias due to deviations from intended interventions	**Moderate**	Patients were blinded and wound care was identical for both halves. However, at least 5000 impulses were applied to 'the wound and the surrounding area', so energy may have spilled over onto the adjacent placebo half (contamination, likely biasing toward the null); no information on adherence to the application protocol or deviations.	*"5000 impulses were set to the wound and the surrounding area."*<br>*"The study participants were also blinded throughout the study…"*
D5 Bias due to missing data	**Moderate**	The number of participants/wound halves analyzed at each time point is not reported; 4/35 did not attend Day 90, and completeness up to Day 12 is implied but not stated. Within-person design means missing data affect both halves equally.	*"Patients completed the treatment (till D90)"*<br>*"Patients who did not show up for the…"*
D6 Bias in measurement of the outcome	**Moderate**	Standardized photographs (tripod, laser distance) with color-based planimetry in Photoshop/ImageJ, i.e. a largely objective measure, applied identically to both halves. Blinding of the image analyst is not clearly stated (the paper says only that the person who allocated was not involved in data evaluation), and the predictable alternation scheme means the allocation could be inferred.	*"…photos were taken in a standardized manner using a reflex…"*<br>*"For the analysis of the collected image data, we used Adobe…"*<br>*"…an investigator who was not involved in the evaluation of the…"*
D7 Bias in selection of the reported result	**Serious**	No registration or protocol is cited. A pre-specified analysis (proportion of patients with complete wound closure per measurement day) is not reported; Day 90 results are reported only as 'no significant difference'; results are given mainly as p-values without dispersion or CIs. Reported values are internally inconsistent (abstract 0.86 vs 0.92 versus results 0.92 vs 0.82 at Day 12; EFD 0.25 mJ/mm2 in Methods vs 0.1 mJ/mm2 in Discussion). Multiple sites, time points and tests create high risk of selective reporting.	*"…ment time and the proportion of patients with complete wound…"*<br>*"…closure per measurement day, calculating the variance and…"*<br>*"…and donor sites (0.86 vs. 0.92, p < 0.05)"*<br>*"0.82% vs. 1B 0.71%; p < 0.01) and Day 12 (0.92 vs. 0.82 p< 0.05)"*<br>*"No significant difference was seen…"*<br>*"0.1 mJ/mm2) on Day 5/7/9 after surgery showed a significantly…"*

Domain	Judgment	Rationale	Supporting quotations
**Overall**	**Serious**	Most severe domain judgment is Serious (D7 selective reporting), with Moderate risk in D1, D4, D5 and D6; overall Serious.	

Yang et al., 2025 [118] — RoB 2

Design as reported: Double-blind, two-parallel-arm, 1:1 randomized clinical trial (sham control). Design as assessed: RCT (parallel). Comparison: Radial ESWT (3 sessions, days 3, 5, 7) + routine dressing treatment vs sham ESWT (device switched off) + routine dressing treatment. Outcome: Percentage wound area reduction ('percentage of wound healing') from day 1 to day 14 (primary outcome; complete closure not reported). Participants: 40 randomized (20 ESWT, 20 RT/sham); 40 analyzed.

Domain	Judgment	Rationale	Supporting quotations
D1 Randomization process	**Some concerns**	The sequence was computer-generated, but allocation concealment is not described. Baseline characteristics (age, sex, cause, burn area, day-3 perfusion) were similar, giving no indication of a problem; under RoB 2, missing information on concealment leads to 'Some concerns'.	*"Eligible patients were randomly assigned (1:1) by a computer…"*<br>*"…generated randomization schedule to receive ESWT (ESWT…"*<br>*"…differences in gender, age, etiology, burn area, or blood…"*
D2 Deviations from intended interventions	**Some concerns**	Sham with the radial device switched off: patients receiving active rESWT feel the impacts while sham patients feel nothing, so participant blinding was probably not effective, and the ESWT operator was not blinded. No information is reported on deviations from the intended interventions arising from the trial context. All 40 randomized patients appear in the analysis. Some concerns, consistent with Taheri et al., 2021 [102] (same switched-off sham, no deviation information).	*"…group, but the shockwave device was turned off."*<br>*"The researcher who applied the ESWT was not blinded to…"*<br>*"Patients and the research, clinical, and laboratory teams were…"*
D3 Missing outcome data	**Low**	Results are reported for 20 vs 20 patients, matching the numbers randomized, and no withdrawals are reported (secondary infection was a pre-defined withdrawal criterion, but no case is described). There is no CONSORT flow diagram in the text.	*"Patients were divided into the RT (n =…"*<br>*"…secondary bacterial infection was a criterion for the with…"*
D4 Measurement of the outcome	**Low**	Wound area was measured with a 3D imaging device (eKare inSight) by a single blinded assessor, identically in both groups.	*"…eKare inSight 3D. The percentage of wound healing was…"*<br>*"…performed by a single, blind assessor."*
D5 Selection of the reported result	**Some concerns**	A ChiCTR registration is cited, but the number (ChiCTR2400…) indicates registration in 2024, after the trial ran from June 2020 to January 2023, i.e. retrospective registration. A retrospective registration cannot protect against selective reporting. Two co-primary outcomes are declared, and Table 3 is internally inconsistent about the statistics used (medians/IQR shown, mean ± SD and t-test stated).	*"…registered in the Chinese Clinical Trial…"*<br>*"Registry (ChiCTR): ChiCTR2400084444."*<br>*"2020 to January 2023."*<br>*"Note: Data is presented as mean ± standard deviation (SD). An independent t-test was used…"*
**Overall**	**Some concerns**	Some concerns in D1, D2, D5 and no domain at high risk, so the overall judgment is Some concerns (standard RoB 2 algorithm; no escalation for multiple Some concerns).	
