# Supplementary material for: Extracorporeal Shock Wave Therapy for Wound Management: Clinical Evidence, Energy Delivery Parameters and Mechanistic Insights—A Systematic Review
Source: J Clin Med. 2026 Nov 3;15(21):8550. doi: 10.3390/jcm15218550 (PMC13763619; doi:10.3390/jcm15218550)
Supplement: Supplementary file 1 [file jcm-15-08550-s001.zip › jcm-4623551_R2 Supplementary File S8.pdf]

PRISMA 2020 Checklist   Manuscript ID: jcm-4623551 Musolf et al.

Section and Topic	Item #	Checklist item	Location where item is reported
**TITLE**			
Title	1	Identify the report as a systematic review.	Title
**ABSTRACT**			
Abstract	2	See the PRISMA 2020 for Abstracts checklist.	Abstract (structured as Background/Objectives, Methods, Results, Conclusions)
**INTRODUCTION**			
Rationale	3	Describe the rationale for the review in the context of existing knowledge.	Section 1 (Introduction)
Objectives	4	Provide an explicit statement of the objective(s) or question(s) the review addresses.	Section 1 (Introduction), final paragraph, objectives (i) to (iv)
**METHODS**			
Eligibility criteria	5	Specify the inclusion and exclusion criteria for the review and how studies were grouped for the syntheses.	Section 2 (Materials and Methods): inclusion criteria (clinical investigations in human subjects evaluating ESWT for wound management, all study designs including reviews, RCTs, controlled cohort studies, uncontrolled case series and case reports) and exclusion criteria (editorials, commentaries, letters to the editor, studies of unrelated clinical conditions, animal and in vitro studies).<br>Grouping for the syntheses: Section 2 with Table 2 and Table 3. Records were grouped by ESWT modality (electrohydraulic, electromagnetic, piezoelectric, radial, modality not disclosed; Tables 5 to 9 and Supplementary Files S2 to S6) and by category of overall outcome (Table 3).
Information sources	6	Specify all databases, registers, websites, organisations, reference lists and other sources searched or consulted to identify studies. Specify the date when each source was last searched or consulted.	Section 2 and Table 1: PubMed and Ovid/Embase, both searched from database inception through September 1, 2026.<br>Section 2: the reference lists of all 46 reviews identified by the database search were screened in addition, on the same date.<br>Figure 2 gives the number of records contributed by each source. No registers, websites or organizations were searched and no study authors were contacted.
Search strategy	7	Present the full search strategies for all databases, registers and websites, including any filters and limits used.	Section 2 and Table 1: the complete search strategy, comprising 12 queries in PubMed and 12 queries in Ovid/Embase, with the number of records retrieved by each. No filters and no language, date or publication-type limits were applied.
Selection process	8	Specify the methods used to decide whether a study met the inclusion criteria of the review, including how many reviewers screened each record and each report retrieved, whether they worked independently, and if applicable, details of automation tools used in the process.	Section 2 and Figure 2: title and abstract screening of the 832 records remaining after removal of 1,878 duplicates, followed by full-text assessment of the 90 publications retrieved and of the publications identified from the reference lists of the 46 reviews, against the eligibility criteria stated in Section 2. Screening of titles and abstracts and full-text assessment were performed independently by two authors (T.W. and C.S.); disagreements were resolved by discussion and, where necessary, by consultation with a third author (C.N.-K.). No automation tools were used.
Data collection process	9	Specify the methods used to collect data from reports, including how many reviewers collected data from each report, whether they worked independently, any processes for obtaining or confirming data from study investigators, and if applicable, details of automation tools used in the process.	Section 2, Table 2 and Supplementary Files S1 to S6: 40 predefined variables were extracted from every included record into a standardized summary by two authors (T.W. and C.S.) independently; disagreements were resolved by discussion and, where necessary, by consultation with a third author (C.N.-K.). Each review was in addition assigned a reference date as defined in Section 2 (Table A1 in Appendix A).<br>Section 2: for articles published in languages other than English, translations were prepared with a large language model to allow assessment of eligibility and data extraction; numerical data extracted from these articles were checked against the tables and figures of the original publications. No automation tools were used for data extraction, and study investigators were not contacted.

Section and Topic	Item #	Checklist item	Location where item is reported
Data items	10a	List and define all outcomes for which data were sought. Specify whether all results that were compatible with each outcome domain in each study were sought (e.g. for all measures, time points, analyses), and if not, the methods used to decide which results to collect.	Section 2 and Table 2: outcomes sought were the overall outcome (V5), the primary endpoint and the time at which it was assessed (V19, V20), the secondary endpoints and their assessment times (V21, V22), and the results reported for the primary and the secondary endpoints (V25, V26). All outcomes reported in a record were extracted as reported, with all measures and timepoints; results are given in Sections 3.2.5, 3.2.17 to 3.2.20, 3.2.22 and 3.2.23 and in Supplementary Files S2 to S6.<br>Section 2 and Section 3.3.1: complete wound healing was the endpoint used for the exploratory quantitative synthesis, together with the criteria a record had to meet to contribute to it.
	10b	List and define all other variables for which data were sought (e.g. participant and intervention characteristics, funding sources). Describe any assumptions made about any missing or unclear information.	Table 2 and Sections 3.2.1 to 3.2.4, 3.2.6 to 3.2.16 and 3.2.24 to 3.2.26: wound type, wound size and duration (V1 to V3), study design (V4), interventions in the ESWT and the control group (V6, V7), group sizes (V8, V9), device and modality (V10, V11; manufacturers in Table 10), energy flux density and its definition (V12, V13), number of shock waves per session and per square centimeter (V14, V15), frequency (V16), number of sessions and interval between them (V17, V18), prospective and retrospective sample size and power analyses (V23, V24, V27, V28), adverse events (V29) and the PEDro items (V30 to V40). Record-level values are given in Supplementary Files S2 to S6.<br>Assumptions about missing or unclear information: Section 2 (reference date of a review where no search date was stated), Section 3.3.4 (publications reporting overlapping participants) and Section 3.3.5 (energy flux densities quoted for radial devices treated as nominal values). Funding sources of the included studies were not extracted.
Study risk of bias assessment	11	Specify the methods used to assess risk of bias in the included studies, including details of the tool(s) used, how many reviewers assessed each study and whether they worked independently, and if applicable, details of automation tools used in the process.	Section 2 and Table 2: methodological quality was appraised with criteria derived from the Physiotherapy Evidence Database (PEDro) scale (variables V30 to V40; cf. [9]). In addition, the risk of bias of every record with a control group was assessed at the domain level by two authors (T.W. and C.S.) independently, with disagreements resolved by discussion, using the Cochrane risk-of-bias tool for randomized trials (RoB 2; crossover variant where applicable) for studies with random allocation and ROBINS-I (version 2) for studies with non-random, including quasi-random, allocation; one outcome was assessed per study (Supplementary File S7). No automation tools were used.<br>Section 4, final paragraph, states that the 25 uncontrolled records could not be appraised with these instruments, because they cannot estimate a comparative effect.
Effect measures	12	Specify for each outcome the effect measure(s) (e.g. risk ratio, mean difference) used in the synthesis or presentation of results.	Section 2, Section 3.3.2 and Table 11: risk ratio with 95% confidence interval for complete wound healing, calculated for each contributing trial and for the pooled estimate.
Synthesis methods	13a	Describe the processes used to decide which studies were eligible for each synthesis (e.g. tabulating the study intervention characteristics and comparing against the planned groups for each synthesis (item #5)).	Section 2: for a dichotomous outcome a record was eligible for pooling only if it reported the number of events together with the number of patients or wounds at risk in both an ESWT and a control group; for a continuous outcome only if it reported means with standard deviations. Where several publications reported the same participants, only one of them entered the quantitative analysis.<br>Section 3.3.1 reports which endpoints met these requirements; Section 3.3.4 identifies the publications with overlapping participants.
	13b	Describe any methods required to prepare the data for presentation or synthesis, such as handling of missing summary statistics, or data conversions.	Section 2: values reported as standard errors, or as medians with quartile ranges, were not converted, because the available conversions rest on distributional assumptions that cannot be verified from the published data. In the one three-arm trial [27] the two active arms were combined and compared with the sham arm.<br>Section 3.3.1 reports how many records fell into each of these categories.
	13c	Describe any methods used to tabulate or visually display results of individual studies and syntheses.	Section 2, Table 2 and Table 3: the standardized extraction of the 40 variables and the categorization of the overall outcome underlying Tables 4 to 9 and Supplementary Files S1 to S6.

Section and Topic	Item #	Checklist item	Location where item is reported
			Displays: Tables 4 to 10 and Table A2 in Appendix A (individual records); Table 11, Table 12 and Figure 3 (synthesis); Figure 4 and Supplementary File S7 (risk of bias); Table A1 in Appendix A (currency of each review); Figure 2 (study selection).
	13d	Describe any methods used to synthesize results and provide a rationale for the choice(s). If meta-analysis was performed, describe the model(s), method(s) to identify the presence and extent of statistical heterogeneity, and software package(s) used.	Section 2: because of the heterogeneity of the included studies a structured qualitative synthesis was chosen as the primary form of analysis, with the rationale given in the same section. For the one endpoint that permitted pooling, risk ratios were combined with a fixed-effect inverse-variance model; no event count was zero, so no continuity correction was applied. Heterogeneity was quantified with Cochran Q and I-squared, tau-squared was estimated by the DerSimonian-Laird method and the random-effects estimate is reported as a check on the fixed-effect result [26]; its confidence interval was additionally calculated with the modified Hartung-Knapp-Sidik-Jonkman method [26]. The calculations were performed with the standard formulae of the inverse-variance method rather than with a dedicated meta-analysis package.<br>Results: Section 3.3.2 and Table 11.
	13e	Describe any methods used to explore possible causes of heterogeneity among study results (e.g. subgroup analysis, meta-regression).	Section 2 and Section 3.3.3: a meta-regression on modality, for which Cochrane guidance recommends at least ten studies, and a subgroup contrast with independently estimable subgroups were both considered and found not to be supported by the available data [26].<br>Section 3.3.5 examines treatment dose as a further potential source of heterogeneity; Section 3.3.2, final paragraph, and Section 4 discuss the clinical and methodological differences between the pooled trials.
	13f	Describe any sensitivity analyses conducted to assess robustness of the synthesized results.	Section 2: three sensitivity analyses were specified, namely exclusion of the trial in which allocation followed the calendar date of referral [23], restriction to the sham-controlled trials [27,28] and exclusion of the trials in which the comparator was hyperbaric oxygen therapy [23,29]; the latter is identical to a restriction to the five randomized trials, because the two HBOT-controlled trials were the two with quasi-random allocation.<br>Results: Section 3.3.2.
Reporting bias assessment	14	Describe any methods used to assess risk of bias due to missing results in a synthesis (arising from reporting biases).	Not assessed. Only one exploratory synthesis, of seven trials, was performed (Section 3.3.2); this does not support a formal assessment of risk of bias due to missing results. The possibility of publication bias in this literature is addressed qualitatively in Section 3.2.27, final paragraph.
Certainty assessment	15	Describe any methods used to assess certainty (or confidence) in the body of evidence for an outcome.	Section 2: the certainty of the evidence for the pooled outcome was rated with the GRADE approach. For the other outcomes, the heterogeneity of definitions and time points did not permit a formal rating (Section 4, final paragraph).
**RESULTS**			
Study selection	16a	Describe the results of the search and selection process, from the number of records identified in the search to the number of studies included in the review, ideally using a flow diagram.	Section 2 and Figure 2: 2,710 records were identified (1,351 in PubMed and 1,359 in Ovid/Embase), 1,878 duplicates were removed, 832 records were screened, 742 were excluded and 90 publications were retrieved; 7 further publications were identified from the reference lists of the 46 reviews, giving 97 records included in the review (51 clinical studies and 46 reviews).<br>Section 3, first paragraph, and Figure 2.
	16b	Cite studies that might appear to meet the inclusion criteria, but which were excluded, and explain why they were excluded.	Section 2 and Figure 2: one publication identified from a reference list (reference 41 in [22]) was excluded because it could not be verified, the link provided redirecting to an unrelated publication and the journal website not listing the article. All other retrieved publications met the inclusion criteria; the records excluded during title and abstract screening did not meet the eligibility criteria stated in Section 2.
Study characteristics	17	Cite each included study and present its characteristics.	The 46 reviews: Section 3.1, Table 4 and Table A1 in Appendix A.<br>The 51 clinical studies: Sections 3.2 and 3.2.1 to 3.2.26, Tables 5 to 9 (by ESWT modality) and Table 10

Section and Topic	Item #	Checklist item	Location where item is reported
			(devices and manufacturers).<br>Supplementary Files S1 to S6 give a standardized summary of every included record, with the 40 variables of Table 2 for each clinical study.
Risk of bias in studies	18	Present assessments of risk of bias for each included study.	Section 3.2.26, Figure 4 and Supplementary File S7: domain-level risk-of-bias judgments (RoB 2 or ROBINS-I) for every controlled record, with the rationale and supporting quotations for every judgment. Section 3.2.26 and Supplementary Files S2 to S6: the PEDro items V30 to V40 for every controlled record.
Results of individual studies	19	For all outcomes, present, for each study: (a) summary statistics for each group (where appropriate) and (b) an effect estimate and its precision (e.g. confidence/credible interval), ideally using structured tables or plots.	Table 11: events and denominators for both groups, with risk ratio and 95% confidence interval, for each of the seven trials contributing to the pooled analysis.<br>Figure 3: proportion with complete wound healing in the ESWT and the control group of all 22 records reporting it, by study design, with events and totals.<br>Sections 3.2.22 and 3.2.23 and Supplementary Files S2 to S6: results for the primary and the secondary endpoints of every record, with the statistical analysis reported by its authors.
Results of syntheses	20a	For each synthesis, briefly summarise the characteristics and risk of bias among contributing studies.	Section 3.3.2 and Table 11: the seven contributing trials with modality, comparator, unit of analysis and assessment timepoint.<br>Section 3.3.2 (risk of bias of the seven contributing trials: some concerns for the five randomized trials, serious risk of bias for the two quasi-randomized trials), Section 3.2.26 and Figure 4 (risk of bias of all controlled records) and Section 3.3.4 (publications reporting overlapping participants, of which only one entered the analysis).
	20b	Present results of all statistical syntheses conducted. If meta-analysis was done, present for each the summary estimate and its precision (e.g. confidence/credible interval) and measures of statistical heterogeneity. If comparing groups, describe the direction of the effect.	Section 3.3.2 and Table 11: complete healing in 155 of 364 patients or wounds treated with ESWT and in 89 of 325 controls; pooled risk ratio 1.51 (95% CI 1.22 to 1.87) in favor of ESWT, based on 689 patients or wounds. Cochran Q was 3.60 on six degrees of freedom, I-squared 0% and tau-squared zero, and the random-effects estimate was identical; the modified Hartung-Knapp-Sidik-Jonkman confidence interval was 1.16 to 1.97.
	20c	Present results of all investigations of possible causes of heterogeneity among study results.	Section 3.3.3 and Table 12: distribution of the controlled and the uncontrolled evidence across the four ESWT modalities, with the reasons why an indirect comparison between them was not possible.<br>Section 3.3.5: the three incompatible dosing conventions and the nominal character of the energy flux densities quoted for radial devices.<br>Section 3.3.2, final paragraph: why the absence of statistical heterogeneity should not be read as agreement between the trials.
	20d	Present results of all sensitivity analyses conducted to assess the robustness of the synthesized results.	Section 3.3.2: excluding the trial in which allocation followed the calendar date of referral [23] gave 1.52 (1.22 to 1.90); restricting the analysis to the two sham-controlled trials [27,28] gave 1.44 (1.10 to 1.88); restricting the analysis to the five randomized trials, which also excludes the two trials in which the comparator was hyperbaric oxygen therapy [23,29], gave 1.44 (1.14 to 1.83).
Reporting biases	21	Present assessments of risk of bias due to missing results (arising from reporting biases) for each synthesis assessed.	Not assessed; see item 14.
Certainty of evidence	22	Present assessments of certainty (or confidence) in the body of evidence for each outcome assessed.	Section 3.3.2: the certainty of the evidence for complete wound healing in DFUs was rated moderate with GRADE (downgraded by one level for risk of bias); the pooled risk ratio corresponds to 140 more healed patients per 1000 (95% CI 60 to 238 more).

Section and Topic	Item #	Checklist item	Location where item is reported
**DISCUSSION**			
Discussion	23a	Provide a general interpretation of the results in the context of other evidence.	Section 4 (Discussion)
	23b	Discuss any limitations of the evidence included in the review.	Section 4, paragraph beginning "Several limitations of the current evidence base should be acknowledged", together with Sections 3.3.1 to 3.3.5.
	23c	Discuss any limitations of the review processes used.	Section 4, final paragraph ("The present review has limitations of its own").
	23d	Discuss implications of the results for practice, policy, and future research.	Section 4 and Section 5 (Conclusions), including the four research priorities set out there.
**OTHER INFORMATION**			
Registration and protocol	24a	Provide registration information for the review, including register name and registration number, or state that the review was not registered.	The review was not registered in PROSPERO or in any other registry (Section 2).
	24b	Indicate where the review protocol can be accessed, or state that a protocol was not prepared.	A review protocol was not prepared in advance (Section 2).
	24c	Describe and explain any amendments to information provided at registration or in the protocol.	Not applicable; the review was not registered and no protocol was prepared.
Support	25	Describe sources of financial or non-financial support for the review, and the role of the funders or sponsors in the review.	Funding statement: "This research received no external funding." No funder or sponsor had any role in the review.
Competing interests	26	Declare any competing interests of review authors.	Conflicts of Interest statement: C.S. served until November 2025 as a consultant for Electro Medical Systems (Nyon, Switzerland), the manufacturer and distributor of one of the devices mentioned in the article, which had no role in study design, data collection and analysis, interpretation of the data or in the decision to publish. No other potential competing interests were reported.
Availability of data, code and other materials	27	Report which of the following are publicly available and where they can be found: template data collection forms; data extracted from included studies; data used for all analyses; analytic code; any other materials used in the review.	Data Availability Statement: the data underlying this article are available in the article and in its online supplementary material.<br><br>Template data collection forms: not applicable; the 40 variables extracted from every record are defined in Table 2.<br><br>Data extracted from included studies: Supplementary Files S1 to S6 (standardized summary of every included record), Supplementary File S7 (risk-of-bias assessments) and Table A2 in Appendix A (which clinical study was cited by which review). Supplementary Files S1 to S6 also publicly available at https://doi.org/10.5281/zenodo.23078056.<br><br>Data used for all analyses: Table 11 (events and denominators of the seven pooled trials), Table 12 and Supplementary Files S2 to S7.

Section and Topic	Item #	Checklist item	Location where item is reported
			Analytic code: not applicable; the pooled estimates were calculated with the standard formulae of the inverse-variance method (Section 2).<br><br>Any other materials used in the review: Table 1 (full search strategy), Table 2 (variable definitions), Table 3 (outcome categorization) and Table A1 in Appendix A (reference date of each review).
